# Root-based inorganic carbon uptake increases the growth of *Arabidopsis thaliana* and changes transporter expression and nitrogen and sulfur metabolism

**DOI:** 10.3389/fpls.2024.1448432

**Published:** 2024-09-06

**Authors:** Liesel Gamarra Reinoso, Imre Majláth, Mihály Dernovics, Attila Fábián, Jeny Jose, Emmanuel Asante Jampoh, Kamirán Áron Hamow, Vilmos Soós, László Sági, Csaba Éva

**Affiliations:** ^1^ Agricultural Institute, Hungarian Research Network (HUN-REN) Centre for Agricultural Research, Martonvásár, Hungary; ^2^ PhD School of Biology, Eötvös Loránd University, Budapest, Hungary; ^3^ Doctoral School of Plant Sciences, Hungarian University of Agriculture and Life Sciences, Gödöllő, Hungary; ^4^ Doctoral School of Horticultural Sciences, Hungarian University of Agriculture and Life Sciences, Gödöllő, Hungary

**Keywords:** anion channel, carbon fixation, homeostasis, root structure, sulfate, transmembrane transporter, nitrate

## Abstract

Root-based uptake of inorganic carbon has been suggested as an additional carbon source. Our study aimed to characterize and understand the root-based uptake and fixation mechanisms and their impact on plant growth. ^13^C-labeled bicarbonate fed to Arabidopsis roots was assimilated into aspartic acid but mainly into sucrose, indicating that the added inorganic carbon was transported to the leaves. A hydroponic treatment was also established for *A. thaliana* using 2 mM NaHCO_3_ at pH 5.6, which enhanced the photosynthetic and growth parameters. According to transcriptome sequencing data, the observed enhancement in growth may be orchestrated by trehalose-6-phosphate signaling and supported by augmented nitrogen and sulfur assimilation. The analysis also revealed regulatory and transporter activities, including several nitrate (*NRT2.1*), and sulfate transporter (*SULTR1;1* and *SULTR1;2*) candidates that could participate in bicarbonate uptake. Different transporters and carbon fixation mutants were assessed. Arabidopsis homologs of SLOW-TYPE ANION CHANNEL 1 (slah3) CARBONIC ANHYDRASE (βca4), and SULFATE TRANSPORTER (sultr1;2) mutants were shown to be inferior to the bicarbonate-treated wild types in several growth and root ultrastructural parameters. Besides, aquaporin genes *PIP1;3* and *PIP2;6* could play a negative role in the carbon uptake by venting carbon dioxide out of the plant. The findings support the hypothesis that the inorganic carbon is taken up by the root anion channels, mostly transported up to the shoots by the xylem, and fixed there by RuBisCo after the conversion to CO_2_ by carbonic anhydrases. The process boosts photosynthesis and growth by providing an extra carbon supply.

## 1 Introduction

Increasing crop yields is a crucial breeding objective, considering global climate change, overpopulation, and the use of crop plants as energy sources. Various studies have suggested that one of the most practical ways to boost crop yield is to increase photosynthetic activity (Éva et al., 2019; Furbank et al., 2020). Promising new research aims to make RuBisCo more specific for CO_2_ (carboxylation reaction) and to increase the speed of the enzyme by improving its kinetics (Gunn et al., 2020; Lin et al., 2022; Zhou et al., 2023). Moreover, several studies have explored ways to enhance CO_2_ supply to RuBisCo in C_3_ plants, such as implementing C_4_ photosynthesis into rice (C_4_ Rice Project) (Von Caemmerer et al., 2012; Karki et al., 2013; Furbank et al., 2023), introducing a photorespiratory bypass within the chloroplast of Arabidopsis (Kebeish et al., 2007), tobacco (South et al., 2019), and rice (Shen et al., 2019), and ectopically over-expressing algal bicarbonate transporters in tobacco (Nölke et al., 2019). Considerable work has been done to better understand leaf CO_2_ uptake and explore possibilities for photosynthetic improvement. However, it has long been known that the rhizosphere contains more CO_2_ than the atmosphere (Nel and Cramer, 2019). Currently, the global average concentration of atmospheric CO_2_ as of June 2024 is 426.91 ppm (http://www.co2.earth/), while Sotomayor and Rice (1999) reported the annual mean soil CO_2_ concentration at a depth of 3 m to be 11000 ppm, Yonemura et al. (2009) reported 1000-23000 ppm at a depth of 10 cm with higher values measured in non-tilled soils. Even at equal levels, CO_2_ uptake in the damp environment of the roots could possibly provide the benefit of less water loss for the plant compared to the leaves with open stomata.

Éva et al. (2019) suggested that root-based inorganic carbon uptake and subsequent transport to shoots would increase photosynthetic fixation. Although the exact mechanism remains unclear, this process has been observed in plants. Different examples of carbon fixation, such as the transport and recycling of root-respired carbon, have been proposed as a means of coping with drought stress (Søndergaard and Sand-Jensen, 1979; Vapaavuori and Pelkonen, 1985; Biatczyk et al., 1994; Bloemen et al., 2013; Dąbrowska-Bronk et al., 2016; Han et al., 2022). Moreover, Dąbrowska-Bronk et al. (2016) confirmed that soluble bicarbonate ions were absorbed from the soil solution by the root hair. As well, it has been demonstrated that increased sucrose concentrations in plants exposed to high CO_2_ levels affect root development. This and other evidence showing ultrastructural changes in the roots of high-carbon-treated plants indicate that root anatomy could play a role in carbon uptake. Similarly, it has been observed that high CO_2_ levels increase the total number, diameter, and length of *Arabidopsis thaliana* roots (Thompson et al., 2017). Furthermore, it has been demonstrated that using lower bicarbonate concentrations 0.7 mM (Vapaavuori and Pelkonen, 1985), 1 mM (Wanek and Popp, 2000), 2 mM (Li et al., 2023), 3 mM (Dąbrowska-Bronk et al., 2016), and 5 mM (Biatczyk et al., 1994) could promote growth, improve intracellular water metabolism, nutrient transport, and photosynthetic capacities. Conversely, higher concentrations such as 6 mM (White and Robson, 1990), 7 and 12 mM (Li et al., 2023), 10 mM (Zribi and Gharsalli, 2002), and 20 mM (Alhendawi et al., 1997) can inhibit plant growth, impair iron acquisition, inhibits effects on plants photosynthetic rate, stomatal conductance, and transpiration rate.

The pH of the solution determines the chemical state of inorganic carbon. At pH 5, most of the inorganic carbon is found in the form of CO_2_, whereas at pH 8, the majority is in the form of bicarbonate ions (Shimono et al., 2019). There are conflicting results regarding the effects of high and low pH values, and further research is required to determine the optimal pH for inorganic carbon uptake. Some studies have shown increased absorption and root-to-shoot transit of inorganic carbon in beans and sunflowers at a low pH (Shimono et al., 2019). For instance, when *A. thaliana* was irrigated with a bicarbonate solution at pH 7 — that is, a high pH solution rich in bicarbonate— it increased plant development (Dąbrowska-Bronk et al., 2016).

Energy-driven uptake of bicarbonate ions is a potential mechanism for root-based inorganic carbon uptake. To the best of our knowledge, true bicarbonate pumps have not yet been isolated from land plants. However, bicarbonate ions were found to inhibit nitrate, sulfate, and phosphate uptake, suggesting a competitive mechanism and indicating that anion transporters with low specificity might also take up bicarbonate (Poschenrieder et al., 2018). Besides the anion transporters, the potential involvement of CO_2_-permeable aquaporins in carbon uptake cannot be ignored, especially at low pH (CO_2_-rich environment). The high protein and sterol content of plant membranes typically act as barriers to CO_2_ diffusion (Groszmann et al., 2017). CO_2_-permeable aquaporins, such as Arabidopsis *AtPIP1;2* and barley *HvPIP2;1*, are highly expressed in the roots (Katsuhara et al., 2002; Postaire et al., 2010). Their function was previously shown to assist CO_2_ in freely crossing membranes and reaching chloroplasts in leaves (Uehleln et al., 2003; Hanba et al., 2004; Heckwolf et al., 2011; Ermakova et al., 2021). Although aquaporins have been shown to play a role in water uptake, their potential contribution to root-based CO_2_ acquisition is yet to be studied (Katsuhara et al., 2002; Postaire et al., 2010).

Following root-based carbon uptake, the plant may fix the carbon or release it back into the environment (Shimono et al., 2019). Carbonic anhydrases (CA) catalyze the reversible conversion between the inorganic carbon forms, which can then serve as a substrate of RuBisCo (CO_2_) or PEP-carboxylase (HCO_3_^-^). All three types of CAs (α-, β-, and γ) are present in plants in various organs (leaf, seed, root) at various subcellular locations (chloroplast, mitochondrion, cytoplasm, plasma membrane), with the β-CAs being the most abundant (DiMario et al., 2017). Traditionally it was thought that the main role of chloroplastic CAs in mesophyll cells of C_3_ plant leaves is to supply RuBisCo with CO_2_ but it found only weak experimental support, except for seedlings (Ferreira et al., 2008). However, CAs in leaves of C_3_ plants take part in various processes like regulation of stomatal movement, biotic and abiotic stress responses, amino acid biosynthesis, and lipid biosynthesis (DiMario et al., 2017; Rudenko et al., 2022). Of the Arabidopsis βCAs, *AtβCA4.2* (located in the cytoplasm) and *AtβCA5* (located in the chloroplast) showed the highest expression in roots (Dimario et al., 2016). A longer isoform (alternative transcript) of *AtβCA4 (AtβCA4.1)* is specifically expressed in the plasma membrane of leaf cells, and together with the apoplastic *αCA2* plays an important part in the transfer of CO_2_ and HCO_3_^−^ to the plant cell (Dimario et al., 2016; Weerasooriya et al., 2024).

PEPC catalyzes the carboxylation of PEP in the presence of HCO_3_^-^ and Mg^2+^ to yield oxaloacetate and inorganic phosphate. In C_4_ and CAM plants, the photosynthetic tissues contain high levels of PEPC that catalyzes the initial fixation of atmospheric CO_2_ during photosynthesis. The much lower levels of PEPC seen in the leaves of C_3_ plants contribute to the enzyme’s anaplerotic function with a key role in the coordination of C and N metabolism, replenishing the TCA cycle with intermediates used for amino acid biosynthesis, and play a role in the regulation of the cellular pH (Miyao and Fukayama, 2003; Nimmo, 2003). It has been described that the fixation of DIC (Dissolved Inorganic Carbon) provided for the roots is associated with the activity of the PEPC (Bialczyk and Lechowski, 1995). Some examples of the PEPC localized in the roots fixing CO_2_ are described byHibberd and Quick (2002) in tobacco and by Brown et al. (2010) in Arabidopsis.

Evidence from the literature supports root-based inorganic carbon uptake, as was previously mentioned. The process behind carbon uptake and fixation by the roots, as well as how it affects plant growth and homeostasis, is still poorly understood. It is also unclear whether carbon is predominantly fixed in the roots and exported to the shoots in an organic form or transported in an inorganic form and fixed in the leaves. Our long-term hypothesis is that our crop plants’ photosynthetic performance and osmotic stress tolerance can be improved by enhancing root-based inorganic carbon uptake. The present study is the first, screening part of the project, aiming to establish a growth-promoting inorganic carbon treatment for Arabidopsis and use it to characterize the root-based uptake, fixation, and growth promotion in terms of anatomy, physiology, and molecular biology. A better understanding of these processes during the present study shall lead to the identification of candidate genes like possible bicarbonate transporter candidates which can be validated and tested in future works and over-expressed in transgenic plants, aiming to boost their photosynthesis and osmotic stress tolerance.

## 2 Materials and methods

### 2.1 Plant material and genotyping

*Arabidopsis thaliana* (ecotype Columbia, Col-0) was used as the plant material for this study. Plants were grown in a reach-in phytotron chamber (Conviron, Winnipeg, Canada) under a 16-h photoperiod (100 μmol m^2^ s^−1^, 21°C/18°C, 75% relative humidity) illuminated with L14 LED tubes with an NS-12 spectral composition (Valoya, Helsinki, Finland). Gene knockout mutants were obtained from the Nottingham Arabidopsis Stock Center (Loughborough, UK). The following mutants were investigated: *Atpip1;2-*1 (Salk_019794C), *Atpip1;2-*2 (Salk_145347C), *Atpip1;3* (Salk_051107C), *Atpip2;6* (Salk_029718C), *βca4* (Salk_067006C), *ppc3* (Salk_143289), *slah3* (Salk_207089C), and *sultr1;2* (Salk_122974). These lines were genotyped, and the segregating lines were bred to homozygosity. The sequences of PCR primers used for genotyping are listed in **Supplementary Table 1**. PCRs were performed on total DNA extracted by the GeneJET Plant Genomic DNA Purification Mini Kit (Thermo Scientific, Waltham, MA, USA). The Phire Plant Direct PCR Master Mix (Thermo Scientific) was used according to the manufacturer’s instructions.

### 2.2 Growth tests

Arabidopsis seeds were surface-sterilized first with 1 ml of 70% (v/v) ethanol (freshly diluted from absolute ethanol supplied by VWR International, Radnor, PA, USA) and then by rotating for 12 min in a mixture of 50% (v/v) ethanol, 1.5% bleach (Feel-It, Egyházasgerge, Hungary), and 0.05% (v/v) Tween 20 (Merck-Sigma Group, Darmstadt, Germany). The seeds were subsequently rinsed with 96% (v/v) ethanol and washed four times with sterile water. Sterile seeds were cold-stratified (4°C) for two days in the dark. The seeds were then germinated, and the plants were grown in solid ½ MS medium (Murashige and Skoog Basal Salt Mixture, pH 5.7; Merck-Sigma Group) with 1% (w/v) sucrose (VWR International) in vertically kept square Petri dishes until they had a well-developed root system (2 weeks) **Supplementary Figure 1A**. In the third week, the treatment started for the standardization of the growth promotion system (**Supplementary Figure 1B**). The plants were transferred to hydroponic cultures supplied with ¼ MS salts together with two different pH: 5.6 and 7.2 and three different concentrations of NaHCO_3_ (Merck-Sigma Group) (0, 2, 4 mM). Once the growth promotion was standardized the mutant’s evaluation came (**Supplementary Figure 1C**), the treatment used was ¼ MS salts at pH=5.6 and 2 mM of NaHCO_3_, which was the system with the best results in the standardization process. The hydroponic system was similar to that described by Tocquin et al. (2003) but with the addition of an aquarium air pump. The hydroponic solution was changed weekly as mentioned in Robison et al. (2006); Conn et al. (2013).

### 2.3 Physiological measurements

General growth indicators, such as root length (RL) and rosette diameter (RD), were measured using a ruler, and total fresh root weight (RW) and total fresh rosette weight (ROW) were measured on a scale Sartorius CP4202S.

### 2.4 Gas exchange analysis

Leaf gas exchange analysis was performed on the third fully developed leaf of 4-week-old plants one day after the last treatment with NaHCO_3_. The CIRAS-3 portable photosynthesis system (PP Systems, Amesbury, MA, USA) was used with a narrow leaf chamber (1.75 cm^2^). CO_2_ fixation rate of control and 2 mM NaHCO_3_ treated plants were investigated at 390 ppm CO_2_ in an infrared gas analysis cuvette, and by maintaining leaf and cuvette temperatures at 21°C. The actinic light was provided by an LED unit of CIRAS 3 (PPFD = 250 µmol m^-2^ s^-1^, RGBW = 60:5:25:10). The net photosynthetic rate (P_n_, µmol CO_2_ m^-2^ s^-1^), stomatal conductance (gs, mmol CO_2_ m^-2^ s^-1^), transpiration rate (E, mmol H_2_O m^-2^ s^-1^), and intracellular CO_2_ concentration (Ci, µmol CO_2_ mol air^-1^) were recorded at the steady-state level of photosynthesis (5-min-lag-time was allowed, and data were recorded at the 15^th^ min of the measurement).

### 2.5 Fluorescence imaging analysis

Chlorophyll-*a* fluorescence induction (FI) analysis was done in the plants grown during the standardization of the growth-promoting system (0, 2, 4 mM NaHCO_3_ and two different pH 5.6 and 7.2). The analysis was carried out using a pulse amplitude modulated fluorometer (PAM) with a blue LED-Array Illumination Unit IMAG-MAX/L (λ=450 nm) (Imaging-PAM MSeries, Walz, Effeltrich, Germany). After 30-min dark adaptation of the plants, the F_0_, F_m,_ and F_v_/F_m_ parameters were determined using a saturation pulse (SP, PPFD = 3000 µmol m^-2^ s^-1^ with 0.8 s duration). The quenching analysis was carried out after a 40-sec lag time at laboratory temperature (adapted state) using continuous blue actinic light (PPFD = 250 µmol m^-2^ s^-1^) and 30-sec SP frequency until the steady-state level of photosynthesis was reached (total duration: 15 min). The actual PSII quantum yield [Y(II)], the quantum yield of the regulated way of energy dissipation [Y(NPQ)], and the quantum yield of the non-regulated way of energy dissipation [Y(NO)] parameters were calculated during analysis, as described by Klughammer and Schreiber (2008).

### 2.6 Fluxomic analysis

For the fluxomic analysis xylem sap and phloem sap were collected from Col plants grown in hydroponic cultures with the standardized growth promotion system (control; 0 mM NaHCO_3_ and treatment; 2 mM NaHCO_3_ and pH 5.6). The plants used were four old weeks and the administration of the labeled carbon (^13^C NaHCO_3,_ Merck-Sigma Group) was added one day before the sample collection. The treatment included three groups: control (not receiving any NaHCO_3_), ^12^C-treated (receiving 2 mM ^12^C NaHCO_3_, purchased from Merck-Sigma Group, carbon composition according to natural abundance: 1.1% ^13^C), and ^13^C-treated (receiving 2 mM ^13^C NaHCO3, purchased from Merck-Sigma Group, containing 99% ^13^C). In the case of the collection of xylem sap, we followed the methodology of Li et al. (2010); and for the phloem sap, we followed the methodology of Guelette et al. (2012).

[1-^13^C^glc^]sucrose (99 atom-% ^13^C; ^13^CC_11_H_22_O_11_) was purchased from Omicron Biochemicals Inc. (South Bend, IN, USA), while DL-aspartic acid-4-^13^C (99 atom-% ^13^C; ^13^CC_3_H_7_NO_4_) and non-labeled sucrose and aspartic acid standards were purchased from the Merck-Sigma Group. Acetonitrile and formic acid (LC-MS grade) were from VWR International. Ultrapure water (18.2 mΩ·cm) was obtained from a Millipore purification system (Merck-Millipore).

From the phloem samples, 300 µL aliquots were evaporated in a vacuum centrifuge and then reconstituted in 300 µL of 9:1 (v/v) acetonitrile: water solution containing 0.5% (v/v) formic acid. The reconstituted samples were vortexed and then centrifuged at 16,000 *g* for 15 min at 4 °C; afterward, 125 µL aliquots were taken from the supernatants and filled into LC-MS vials equipped with microinserts. From the xylem samples, 15 µL aliquots were diluted with 135 µL of acetonitrile containing 0.55% (v/v) formic acid. The samples were vortexed and then centrifuged at 16,000 *g* for 15 min at 4 °C; afterward, 100 µL aliquots were taken and handled as above.

To determine the multiple reaction monitoring (MRM) transitions for the quantification of labeled and non-labeled analyses, ESI-MS/MS analysis was carried out using an Acquity I-class ultra-performance liquid chromatography (UPLC) system coupled to a Xevo TQ-XS Triple Quadrupole Mass Spectrometer (Waters, Milford, MA, USA), used in electrospray ionization (ESI) negative mode. Separation was performed on a ZIC-cHILIC column (Merck; 100 mm * 2.1 mm * 3.0 µm) at 40 °C. For gradient elution, water and acetonitrile containing 0.5% (v/v) formic acid were used. The applied gradient and the UPLC-ESI-MS/MS parameters (**Supplementary Table 2**) are presented together with the relevant MS/MS spectra (**Supplementary Figures 2**-**6**). For the selective quantification of non-labeled and [1-^13^C^glc^]-labeled sucrose molecules, the MRM transitions of *m/z* 341→161 and *m/z* 343→163 were used, respectively. The intensity values of *m/z* 343→163 (‘A+1’ isotopolog for [1-^13^C^glc^] sucrose in ESI negative mode) determined for the samples in the labeling experiment were corrected with the residual *m/z* 343→163 data of non-labeled sucrose standard (‘A+2’ isotopolog for natural sucrose in ESI negative mode).

For the selective quantification of natural aspartic acid and aspartic acid-4-^13^C molecules, the MRM transitions of *m/z* 132→88 and *m/z* 133→88 were used, respectively. The intensity values of *m/z* 133→88 (‘A’ isotopolog for aspartic acid-4-^13^C in ESI negative mode) determined for the samples in the labeling experiment were corrected with the residual *m/z* 133→88 data of non-labeled aspartic acid standard (‘A+1’ isotopolog for natural aspartic acid in ESI negative mode).

### 2.7 Light microscopy

The required chemicals were from Merck-Sigma Group, unless otherwise stated. For root anatomy, the following genotypes were evaluated Col, *pepc3, βca4, sultr1;2, slah3, pip1;2-1, pip1;2-2* and the treatment was 0 mM or 2 mM NaHCO_3_ the samples were obtained from the root differentiation zone with root hairs. The roots were fixed in 50 mM Na-cacodylate buffer (pH 7.2) containing 2.5% (v/v) glutaraldehyde, and 4% (v/v) formaldehyde overnight at 4°C, washed, dehydrated in an ethanol series, and gradually infiltrated with London Resin (LR) White acrylic resin (Ted Pella, Redding, CA, USA) according to the manufacturer’s instructions. The resin was polymerized under UV light at -20°C. Semi-thin sections (1 μm) were serially sectioned at the transverse plane of the roots using an Ultracut-E microtome (Reichert-Jung, Heidelberg, Germany) and stained with periodic acid-Schiff (PAS) and 1% (w/v) Amido Black for polysaccharides and proteins, respectively. The stained sections were mounted in 50% (v/v) glycerol containing 7% (v/v) acetic acid and examined under a BX51 light microscope (Olympus, Tokyo, Japan). Photographs were captured using a TrueChrome-II color camera (Tucsen Photonics, Gaishan Town, Fujian, PR China) with integrated software to measure the parameters of root diameter (RTD), epidermis width (EpWd), endodermis width (EnWd), cortex width (CxWd), stele diameter (StDm) and outer cortical cell width (OuCCWd). The anatomy and various parts of the Arabidopsis roots were identified according to Buchner et al. (2004).

### 2.8 Transcriptome sequencing

Samples from middle-aged rosette leaves and roots of approximately 4 weeks old plants were collected at the end of the bicarbonate treatment. For total RNA extraction, 70 mg of each sample in three repetitions were homogenized in 500 μL of TRI-Reagent (Zymo Research, Irvine, CA, USA). Total RNA was extracted using the Direct-zol RNA MiniPrep System (Zymo Research), according to the manufacturer’s protocol, including the on-column DNase digestion. IRNA concentrations were measured using a NanoDrop 2000 Spectrophotometer (Thermo Scientific), and the A260/A280 ratio was used to estimate the RNA quality. Total RNA samples were then subjected to quality control and custom sequencing. All our samples passed the quality control and had an RNA integrity number (RIN) of ≥7. The general parameters of the analysis included the production of 150-bp pair-end reads and 12 Gbp raw data per sample on the Illumina NovaSeq 6000 platform (Novogene, Nanjing, China), and the reads were mapped to the ensemblplants_arabidopsis_thaliana_tair10_gca_000001735_1 genome assembly. Only differentially expressed genes (DEGs) with significant (*p*<0.05) and log_2_FC ≥ |1| were involved in any further investigation, including gene ontology (GO) enrichment (Ashburner et al., 2000) and Kyoto Encyclopedia of Genes and Genomes [KEGG; (Kanehisa and Goto, 2000)] pathway (org code: ath) analysis. These functional analyses have been implemented by the clusterProfiler R package, in which gene length bias was corrected. The graphics for the volcano plots were done using Venny 2.1 (https://bioinfogp.cnb.csic.es/tools/venny/index.html), the heatmaps were prepared with Heatmapper (http://heatmapper.ca/), and the bar graphics and the enrichment bubble plot with SRplot (https://www.bioinformatics.com.cn/en). Four treatments are reflected in the analysis, the MSRC (Murashige and Skoog Root Control), MSRT (Murashige and Skoog Root Treated with the 2mM NaHCO_3_), MSLC (Murashige and Skoog Leaf Control), MSLT (Murashige and Skoog Leaf Treated with the 2mM NaHCO_3_).

### 2.9 qPCR validation of the RNAseq data

Total RNA was extracted in the same way as described above. The samples (1,000 ng in each reaction) were then reverse-transcribed using random primers and the RevertAid First Strand cDNA Synthesis Kit (Thermo Scientific) according to the manufacturer’s instructions. The obtained cDNA was diluted threefold and used as a template for qPCRs. The reactions were run in triplicate using the CFX96 Touch Real-Time PCR Detection System (Bio-Rad, Hercules, CA, USA) and the PCRBIO SyGreen Mix (PCR Biosystems, London, UK).

Several transporter candidates and genes participating in cell wall composition were amplified, and their expression was compared between qPCRs and transcriptome sequencing. The transcription levels of the studied genes were calculated using the 2^-ΔΔCt^ method (Livak and Schmittgen, 2001) with an efficiency correction step applied according to (Pfaffl, 2004). Primers used for qPCR validation are listed in **Supplementary Table 3**.

### 2.10 Statistical analysis

For the optimization of the growth promotion system, sample number (no. of plants measured) for the growth parameters was n=40, for the gas exchange analysis n=4, and for the fluorescence imaging n=10. This was individual for each of the group treatments of the pH: 5.6 and pH 7.2, with the respective treatments 0mM (control), 2mM and 4mM NaHCO_3_. The data is shown as mean ± SD and were statistically analyzed using a two-way ANOVA testing the effect of pH and the concentration of bicarbonate on the given growth or photosynthetic parameter followed by Tukey’s HSD *post-hoc* test in RStudio 4.3.0.

For the evaluation of the mutant plants, sample number (no. of plants measured) for the growth parameters was n=12, for the gas exchange n=4, for the fluorescence imaging n=12 and for the light microscopy evaluation n=12. This is for each of the mutants evaluated with the corresponding treatment (0mM and 2mM). An independent sample Student’s *t*-test (*p*<0.05) was performed for the comparison of the treatments per genotype in Microsoft Excel. Data are shown as the mean ± SD.

For the fluxomics analysis, six samples were evaluated using an independent sample Student’s *t*-test (*p*<0.05) to compare the control and the treatment with labeled and non-labeled 2 mM NaHCO_3_ and pH 5.6.

## 3 Results

### 3.1 Optimization of growth-promoting inorganic carbon treatment for *A. thaliana*

Evidence from the literature suggests that low-concentration bicarbonate solutions have growth-promoting effects. Our experiments began with the growth optimization of hydroponic treatment in *Arabidopsis thaliana* (Col). Low (2 mM) and high (4 mM) NaHCO_3_ treatments were tested at different pHs (5.6 and 7.2). We observed growth promotion (compared to untreated plants) at a 2 mM pH 5.6 NaHCO_3_ condition. It improved the root and rosette weight and rosette diameter. Additionally, the net photosynthesis rate increased significantly at 2 mM NaHCO_3_ and pH 5.6 (see **Supplementary Figures 7**-**9**). On the other hand, high-concentration (4 mM) bicarbonate treatments were inhibitory, especially at high pH to the point that gas exchange parameters could not be determined due to the state of the plants.

### 3.2 Uptake and fate of inorganic carbon supplied to roots

**Figure 1:**
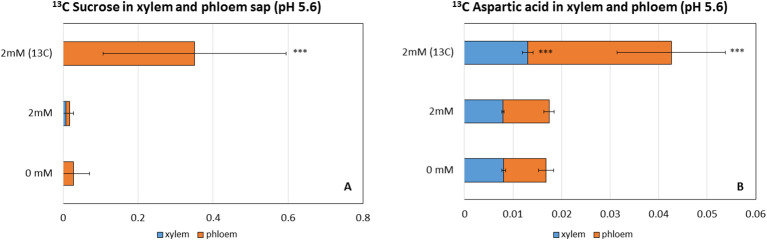
The concentration of aspartic acid and sucrose (nM) in the xylem and phloem sap of plants treated with 0mM, ^12^C 2 mM NaHCO_3_ and ^13^C 2 mM NaHCO_3_ as sources of inorganic carbon applied to the roots. **(A)** Level (nM) of ^12^C, ^13^C aspartic acid in the xylem and phloem sap, **(B)** Level (nM) of ^12^C, ^13^C sucrose in the xylem and the phloem sap. Each value represents the mean ± SD of n=6. Asterisks indicate statistically significant differences (p-value ≤0.05*, ≤0.01 **, ≤0.001 ***, Paired Student t-test).

A fluxomic study was performed using carbon isotope (^12^C and ^13^C)-labeled NaHCO_3_ to monitor the uptake of inorganic carbon and fixation of the derivative carbon in wild-type plants grown in the optimized, growth-promoting pH 5.6 hydroponic solution containing 2 mM NaHCO_3_. Aspartic acid, which can be produced by the transamination of oxaloacetate catalyzed by PEPC, was used as a marker for PEPC-based carbon fixation. Whereas sucrose is the main photoassimilate in land plants and can be considered an indirect marker of RuBisCo-based carbon fixation. The analysis revealed that ^13^C-labeled sucrose could not be detected in the xylem sap (**Figure 1A**). However, 0.06 nM of ^13^C of aspartic acid was found in the xylem sap (**Figure 1B**) of plants treated with ^13^C, a three-fold increase compared to the control. In contrast to the xylem sap, but in line with the physiological function of the phloem, a considerable amount of ^13^C-labeled sucrose was detected in the phloem sap (1.02 nM, a 20-fold increase in the concentration as a result of the ^13^C treatment) (**Figure 1A**), suggesting that NaHCO_3_ was taken up by the roots and the carbon was assimilated and incorporated into sucrose. The treatment also increased the ratio of ^13^C sucrose compared to the ^12^C one by 12-fold, reaching 2% of all sucrose (**Supplementary Figure 10**). Similarly, a 4-fold higher level of ^13^C-labeled aspartic acid (0.22 nM) was detected in the phloem sap of treated plants, (**Figure 1B**), but its ratio reached only 0.4% of all aspartic acids.

Therefore, we proved the uptake and fixation of the hydroponically supplied carbon in Col during the optimized 2 mM NaHCO_3_ treatment at pH 5.6, and we mostly found fixed carbon in sucrose in the phloem sap.

### 3.3 Transcriptional responses to NaHCO_3_ treatment in roots and leaves in Arabidopsis plants

Although numerous transporters and channels are thought to be associated with the uptake and translocation of inorganic carbon, it is still unclear whether they are truly involved in CO_2_/HCO_3_^-^ uptake by roots and transport in plants. To identify more candidate genes involved in CO_2_/HCO_3_^-^ translocation and to better understand the mechanism of fixation and the effect of carbon uptake, we performed RNA-Seq analysis of the roots and rosette leaves of 4-week-old wild-type Col-0 plants grown under control and 2 mM NaHCO_3_ regimens in triplicate.

**Figure 2:**
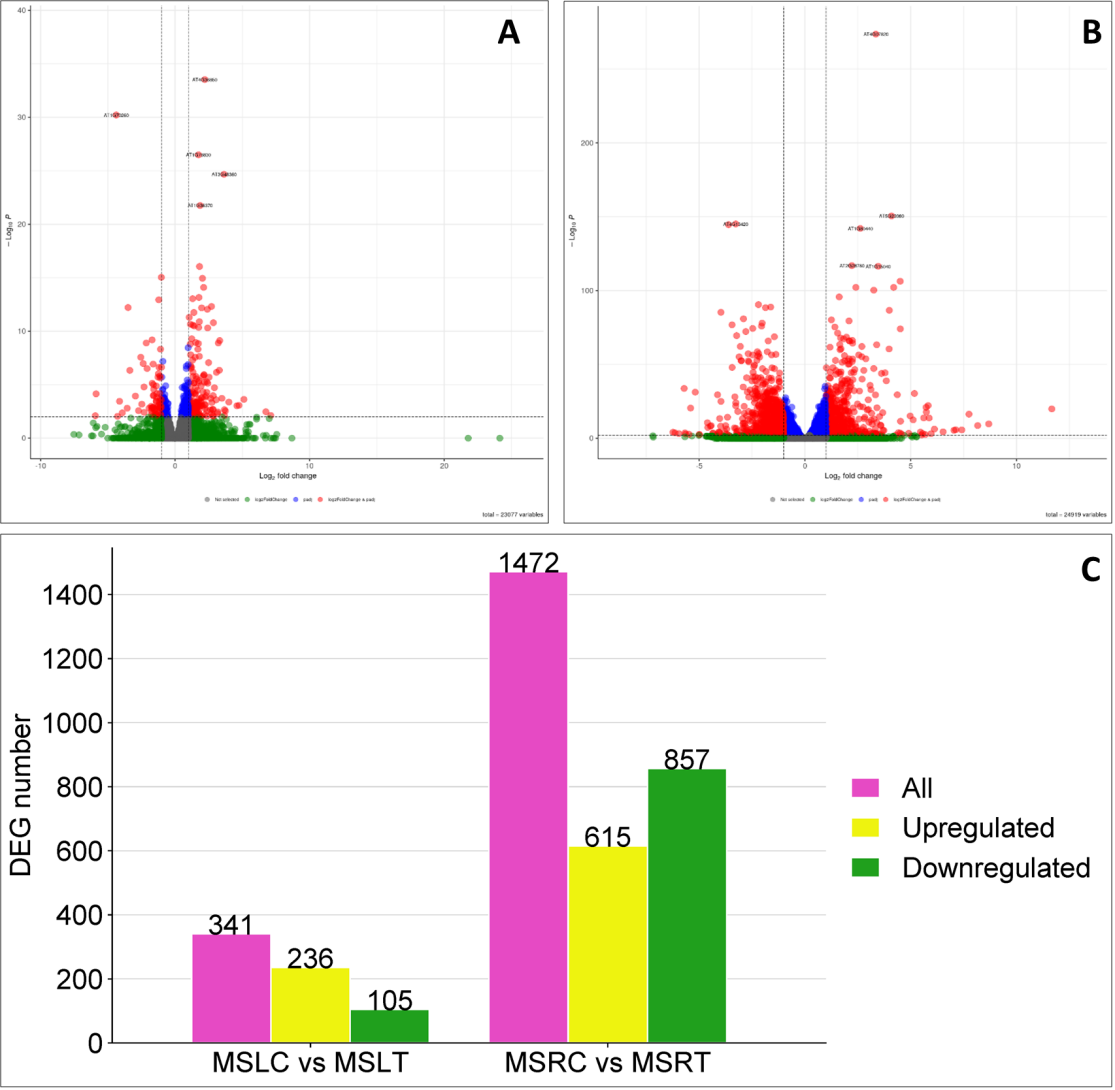
Volcano plots and illustration of differentially expressed genes in MSLC vs MSLT and MSRC vs MSRT. **(A)** Representation of the volcano plot for the MSLC vs MSLT. **(B)** Representation of the volcano plot for the MSRC vs MSRT. **(C)** The total, the downregulated, and the upregulated DEGs are presented in the MSLC vs MSRT and MSRC vs MSRT. The volcano plots and the bar representation were graphic considering a based Log_2_ Fold change ≥ 1/-1 and P-adj ≤ 0.05.

Based on log2FC≥ 1 (upregulation), Log_2_FC≤-1 (downregulation) and *p-*adj ≤ 0.05, we identified 341 genes that were differentially expressed in the leaves of the control and treated plants. The number of differentially expressed genes (DEGs) that were upregulated in the leaves was 236 and downregulated by 105 (**Figures 2A, C**). In the roots, 1472 genes were differentially expressed after bicarbonate treatment. The number of upregulated DEGs was 615 and 857 were downregulated (**Figures 2B, C**). Overall, more genes were affected in the roots than in leaves.

**Figure 3:**
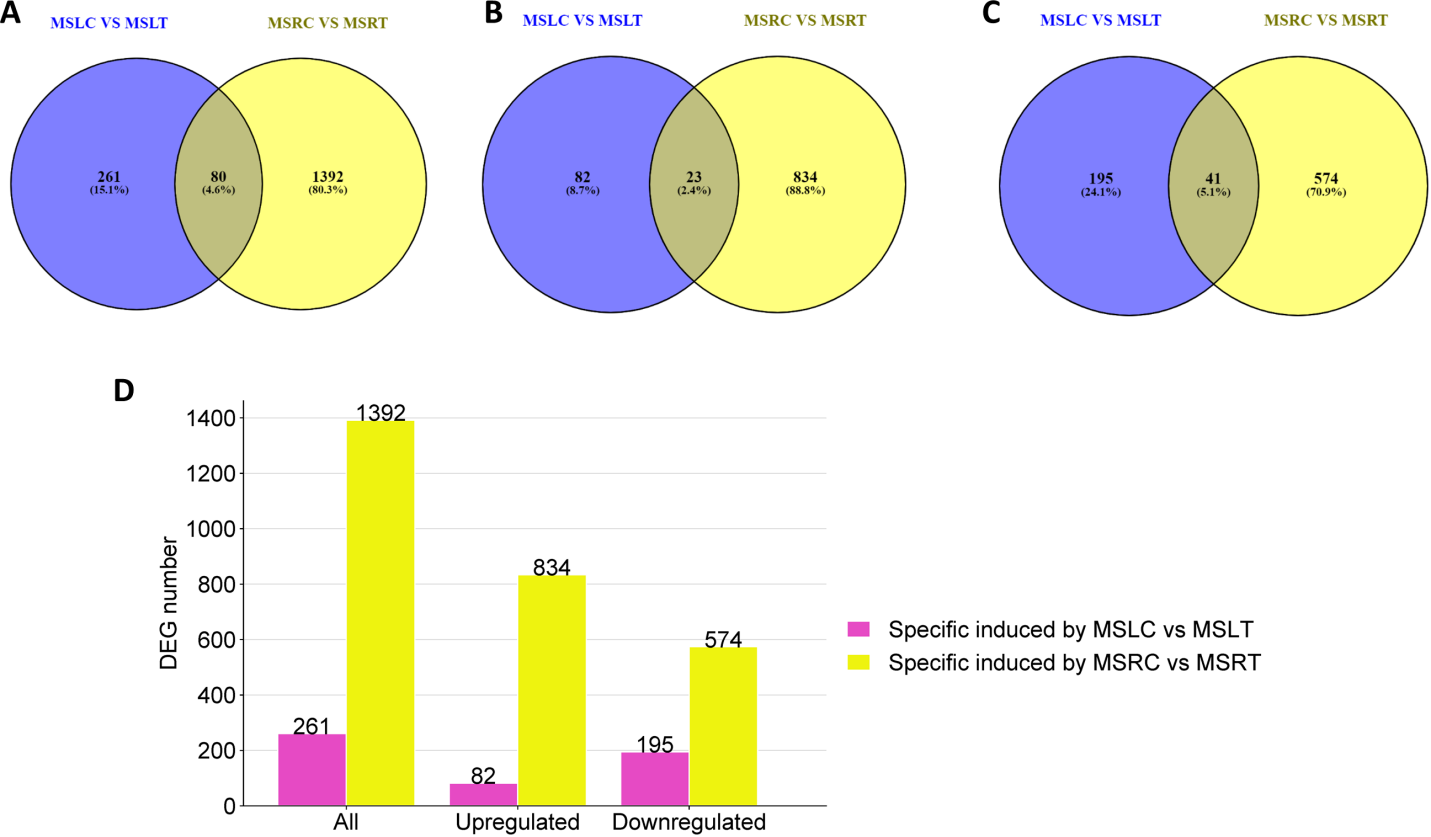
Venn diagrams and bar plot comparing MSLC vs. MSLT and MSRC vs. MSRT. A Venn diagram of all the DEGs present in MSLC vs MSLT and MSRC vs MSRT. **(B)** Venn diagram of upregulated DEGs present in the MSLC vs MSLT and MSRC vs MSRT. **(C)** Venn diagram of downregulated DEGs present in the MSLC vs MSLT and MSRC vs MSRT. **(D)** The number of DEGs specifically regulated by MSLC vs MSLT and MSRC vs MSRT.

Venn diagrams were used to investigate the presence of shared DEGs between the roots and leaves of control and treated plants. When comparing the DEGs reported for MSRC vs. MSRT and MSLC vs. MSLT (**Figure 3A**), only 80 DEGs were present in both roots and leaves. Furthermore, the results indicated that only 23 and 41 DEGs were commonly upregulated and downregulated, respectively, in the roots and leaves (**Figures 3B, C**). Additionally, 261 DEGs were exclusively present in MSLC vs. MSLT, with 82 upregulated and 195 downregulated genes (**Figure 3D**). For MSRC vs. MSRT, 1392 DEGs were expressed, with 834 upregulated and 574 downregulated genes (**Figure 3D**). A heatmap representation of the comparison of DEGs between MSLC, MSLT, MSRC, and MSRT is provided in **Supplementary Figure 11**.

**Figure 4:**
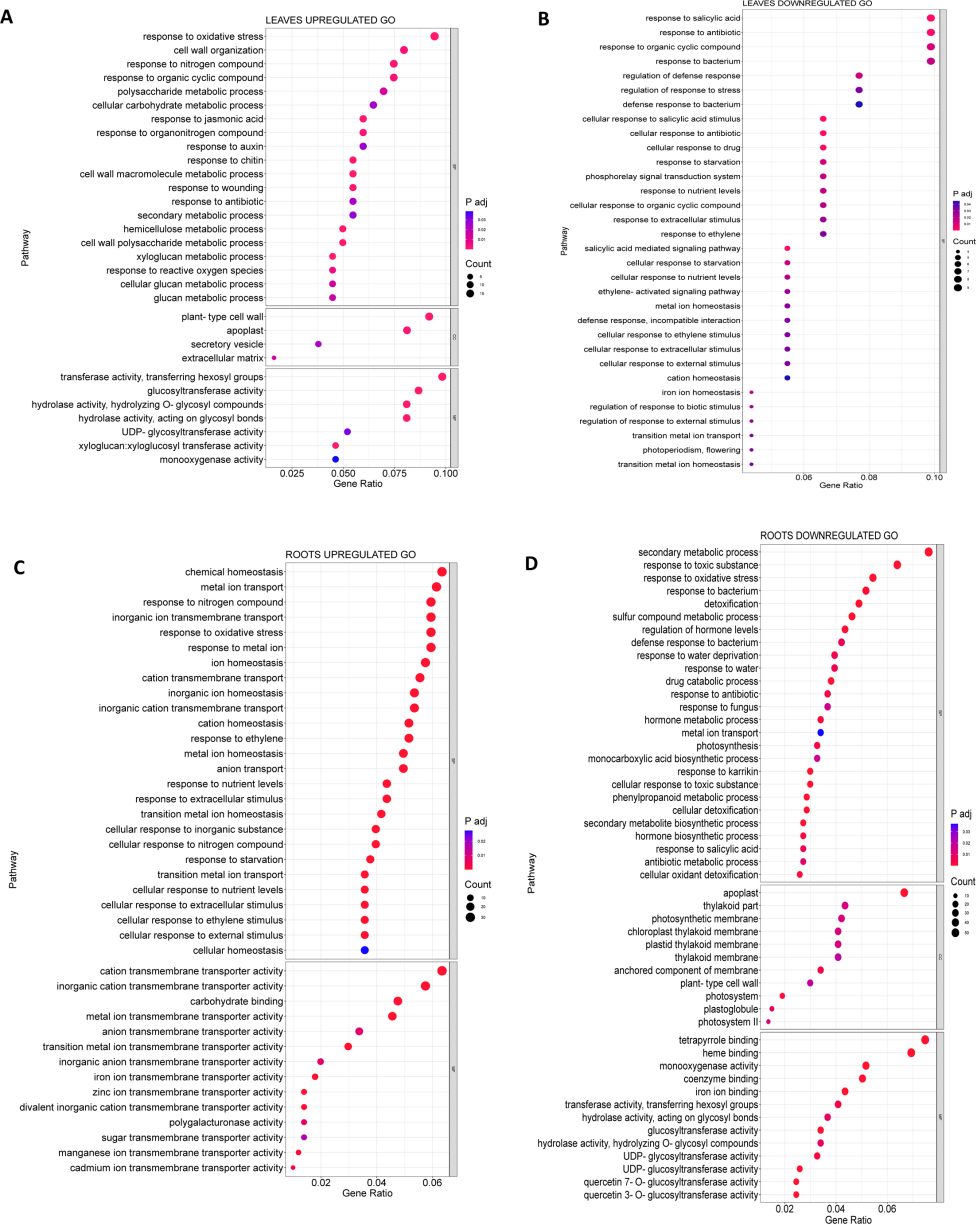
GO analysis for the leaves and the roots. **(A)** Upregulated GO terms for MSLC vs MSLT. **(B)** Downregulated GO terms for the MSLC vs MSLT. **(C)** Upregulated GO terms for the MSRC vs MSRT. **(D)** Downregulated terms for the MSRC vs MSRT.

To further investigate the function of the DEGs regulated in MSLC vs. MSLT and MSRC vs. MSRT, Gene Ontology (GO) and Kyoto Encyclopedia of Genes and Genomes (KEGG) analyses were carried out. According to the GO analysis, 80 GO terms were assigned to the MSLC vs. MSLT group, while a total of 237 GO terms were significantly observed in the MSRC vs. MSRT group. There were 37 GO terms assigned to MSLC vs. MSLT and MSRC vs. MSRT. Some of the common GO terms were response to nitrogen compound (GO:1901698), trehalose biosynthetic process (GO:0005992), and response to starvation (GO:0042594). From upregulated DEGs, 49 GO terms were assigned to the leaves and 92 GO terms were assigned to the roots, of which three GO terms were common between the two: response to nitrogen compound (GO:1901698), response to reactive oxygen species (GO:0000302), and response to oxidative stress (GO:0006979). For the downregulated DEGs, 48 GO terms were assigned to the leaves, and 168 GO terms were assigned to the roots. Among the downregulated GO terms, nine were common to the leaves and roots, notably e.g. iron ion transport (GO:0006826) and cutin biosynthetic process (GO:0010143), (GO terms can be found in **Supplementary Tables 4**-**7**). **Figure 4** shows the GO terms with a higher number of genes.

For the upregulated MSLC vs. MSLT (**Figure 4A**), the GO terms with the most genes for BP (biological process) were response to oxidative stress (GO:0006979), cell wall organization (GO:0071555), response to nitrogen compound (GO:1901698), and response to organic cyclic compound (GO:0014070). For the CC (cellular component) plant-type cell wall (GO:0009505) and apoplast (GO:0048046). MF (molecular function) showed transferase activity, transferring hexosyl groups (GO:0016758), and glucosyltransferase activity (GO:0046527). For downregulated MSLC vs. MSLT (**Figure 4B**), only GO terms related to BP were obtained, such as response to organic cyclic compound (GO:0014070).

For the roots, the upregulated GO terms (**Figure 4C**) for BP were chemical homeostasis (GO:0048878), metal ion transport (GO:0030001), response to nitrogen compound (GO:1901698), and inorganic ion transmembrane transport (GO:0098660). For MF cation transmembrane activity (GO:0008324), and inorganic cation transmembrane transporter activity (GO:0022890). Interestingly, many GO terms related to transmembrane transporter activity, homeostasis, and cellular responses to stimuli were upregulated in the roots. The downregulated GO terms for the roots (**Figure 4D**) of BP were secondary metabolic process (GO:0019748), response to toxic substance (GO:0009636), and response to oxidative stress (GO:0006979). In contrast to the leaves, the presence of the GO term for the apoplast (GO:0048046) was downregulated. MF terms related to tetrapyrrole binding (GO:0046906) and heme binding (GO:0020037) contained the highest downregulated number of DEGs.

**Figure 5:**
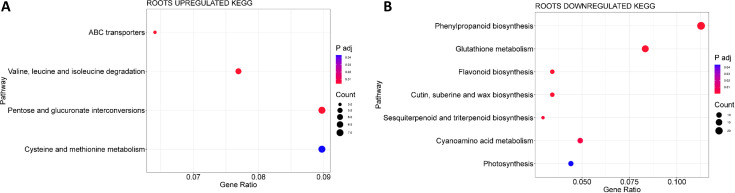
KEGG analysis for the MSRC vs MSRT. **(A)** Upregulated KEGG pathways. **(B)** Downregulated KEGG pathways.

KEGG analysis for the MSLC vs. MSLT and MSRC vs. MSRT comparisons identified one upregulated KEGG pathway (Cutin, suberin, and wax biosynthesis, ath00073) and nine downregulated KEGG pathways, respectively. Cutin, suberin, and wax biosynthesis was present in both the roots and leaves. In the leaves, starch and sucrose metabolism (ath00500) was upregulated and cutin, suberin, and wax biosynthesis (ath00073) was downregulated (**Supplementary Tables 8**; **9**). DEGs present in the upregulated pathway were TPS9/TPS8/TPS10/TPS11 (trehalose phosphate synthases), which are involved in carbon metabolism under stress (Usadel et al., 2008; Ponnu et al., 2011). Additionally, some BGLU (β- glucosidases) were found, these are involved in many processes of plant secondary metabolism as well are known to be intermediaries in cell wall composition, phytohormone activation, and the activation of chemical defense compounds (Wang et al., 2022). The BGLUs found were BGLU16 which is involved in lignin synthesis and cell wall formation and BGLU23 which participates in endoplasmic reticulum (ER) body formation and function (Yamada et al., 2020; Sarkar et al., 2021). The DEGs in the downregulated pathway were cytochrome P450-dependent oxidases CYP86A7/CYP77A6, which participated in cutin biosynthesis (Mazurek et al., 2017) and FAR8, which are involved in the fatty acid composition of cell walls (Chacón et al., 2013). In the roots, four KEGG pathways were upregulated (**Figure 5A**; **Supplementary Table 10**): cysteine and methionine metabolism (ath00270); pentose and glucuronate interconversions (ath00040); valine, leucine, and isoleucine degradation (ath00280); and ABC transporter (ath02010). Seven KEGG pathways were downregulated (**Figure 5B**; **Supplementary Table 11**), including phenylpropanoid biosynthesis (ath00940), glutathione metabolism (ath00480), cyanoamino acid metabolism (ath00460), and cutin, suberin, and wax biosynthesis (ath00073).

Most DEGs upregulated in the roots were related to transmembrane transport activity and homeostasis. We then decided to look forward to the presence of all transporters and channels. **Tables 1**, **2** list the up-regulated and down-regulated transporters and channels, respectively. We observed that sugar, ABC, vacuolar iron, and phosphate transporters were the families with the most up-regulated DEGs. The nitrate transporter *NRT2.1*, together with the zinc transporter *ZIP10*, showed the highest fold-change; however, the expression of *ZIP10* was not high. Similar to this upregulation, some ABC and sugar transporters were downregulated.

**Table 1:** Transporters and channels upregulated in the MSRC vs. MSRT.

Family	Gene ID	Gene Description	Gene Name	Log2 FC ≤ 1	FC	Gene expression RT
Zinc transporter	AT1G31260	Probable zinc transporter 10	*ZIP10*	4.57	23.73	3.84
	AT1G05300	Zinc transporter 5	*ZIP5*	1.35	2.55	247.31
Nitrogen transporter	AT1G08090	High-affinity nitrate transporter 2.1	*NRT2.1*	3.53	11.54	515.89
Vacuolar iron transporter	AT3G43630	Vacuolar iron transporter homolog 3	*-*	3.35	10.19	29.12
	AT3G25190	Vacuolar iron transporter homolog 2.1	*-*	1.27	2.41	8816.93
	AT1G76800	Vacuolar iron transporter homolog 2	*-*	1.19	2.28	1148.67
	AT1G21140	Vacuolar iron transporter homolog 1	*-*	1.18	2.26	1957.39
Aluminum transporter	AT4G00910	Aluminum-activated malate transporter family protein	*-*	3.25	9.53	1151.43
Sugar transporter	AT5G53190	Bidirectional sugar transporter SWEET	*SWEET3*	2.84	7.16	31.40
	AT3G48740	Bidirectional sugar transporter SWEET11	*SWEET11*	2.83	7.13	29.29
	AT5G23660	Bidirectional sugar transporter SWEET12	*SWEET12*	2.75	6.71	592.31
	AT3G16690	Bidirectional sugar transporter SWEET	*SWEET16*	1.03	2.04	727.87
ABC transporter	AT3G28360	ABC transporter B family member 16	*ABCB16*	2.56	5.89	88.79
	AT4G28620	ABC transporter B family member 24, mitochondrial	*ABCB24*	2.11	4.32	17.66
	AT4G01820	ABC transporter B family member 3	*ABCB3*	1.73	3.33	69.49
	AT4G01830	ABC transporter B family member 5	*ABCB5*	1.58	2.99	14.27
	AT3G21090	ABC transporter G family member 15	*ABCG15*	1.40	2.65	246.46
	AT3G28380	ABC transporter B family member 17	*ABCB17*	1.32	2.49	61.22
	AT1G10680	ABC transporter B family member 10	*ABCB10*	1.10	2.14	116.26
Nucleotide transporter	AT1G02630	Equilibrative nucleotide transporter 8	*ETN8*	2.44	5.41	18.95
Sulfate transporter	AT4G08620	Sulfate transporter 1.1	*SULTR1;1*	1.84	3.57	1319.15
Phosphate transporter	AT5G43370	Probable inorganic phosphate transporter 1-2	*PHT1-2*	1.63	3.10	201.71
	AT3G23430	Phosphate transporter PHO1	*PHO1*	1.55	2.92	26587.58
	AT2G32830	Probable inorganic phosphate transporter 1-5	*PHT1-5*	1.50	2.82	13.88
	AT4G25350	Phosphate transporter PHO1 homolog 4	*PHO1-H4*	1.18	2.27	316.38
Sodium transporter	AT4G10310	Sodium transporter HKT1	*HKT1*	1.46	2.75	2566.46
Molybdate transporter	AT2G25680	Molybdate transporter 1	*MOT1*	1.41	2.66	405.74
Carnitine transporter	AT1G79360	Organic cation/carnitine transporter 2	*OCT2*	1.37	2.59	218.72
	AT1G16390	Organic cation/carnitine transporter 3	*OCT3*	1.09	2.13	642.40
Aminoacid transporter	AT5G40780	Lysine histidine transporter 1	*LHT1*	1.12	2.17	5278.25
Magnesium transporter	AT1G29830	Magnesium transporter CorA-like family protein	*-*	1.10	2.14	83.20
Potassium transporter	AT5G14880	Potassium transporter	*POT8*	1.09	2.13	5594.82
Metal transporter	AT1G80830	Metal transporter Nramp1	*NRAMP1*	1.02	2.03	8881.67
Anion Channel	AT5G24030	S-type anion channel SLAH3	*SLAH3*	1.41	2.67	8093.20
Ion transporter	AT5G14870	Cyclic nucleotide-gated ion channel 18	*CNGC18*	1.25	2.38	64.22
Aquaporin channels	AT5G60660	PIP2F	*PIP2-4*	1.03	2.05	6700.77

FC, Fold change; RT, Treated root.

**Table 2:** Transporters and channels downregulated in the MSRC vs. MSRT.

Family	Gene ID	Gene Description	Gene Name	Log2 FC ≤ -1	FC	Gene expression RT
ABC transporter	AT3G59140	ABC transporter C family member 10	*ABCC10*	-1.01	0.50	180.78
	AT5G44110	ABC transporter I family member 21	*ABCI21*	-1.04	0.49	530.35
	AT3G53510	ABC transporter G family member 20	*ABCG20*	-1.21	0.43	1007.02
	AT3G25620	ABC transporter G family member 21	*ABCG21*	-1.23	0.43	24.26
	AT1G02520	ABC transporter B family member 11	*ABCB11*	-1.40	0.38	2231.45
	AT3G55090	ABC transporter G family member 16	*ABCG16*	-1.41	0.38	1093.46
	AT3G47780	ABC transporter A family member 7	*ABCA7*	-1.48	0.36	1765.55
	AT5G06530	ABC transporter G family member 22	*ABCG22*	-1.52	0.35	20.79
	AT3G30842	ABC transporter G family member 38	*ABCG38*	-1.68	0.31	45.14
	AT5G52860	ABC transporter G family member 8	*ABCG8*	-1.68	0.31	16.38
	AT5G19410	ABC-2 type transporter family protein	*-*	-2.08	0.24	250.66
	AT5G13580	ABC transporter G family member 6	*ABCG6*	-2.11	0.23	941.00
	AT3G13100	ABC transporter C family member 7	*ABCC7*	-1.57	0.34	993.31
Sugar transporter	AT4G25010	Bidirectional sugar transporter SWEET	*SWEET14*	-1.02	0.49	13.49
	AT3G05400	Sugar transporter ERD6-like 12	*SUGTL5*	-1.19	0.44	43.19
	AT1G12600	UDP-galactose/UDP-glucose transporter 4	*UTR4*	-1.04	0.49	86.57
	AT3G05400	Sugar transporter ERD6-like 12	*SUGTL5*	-1.19	0.44	43.19
Phosphate transporter	AT1G69480	Phosphate transporter PHO1 homolog 10	*PHO1-H10*	-1.04	0.49	31.46
Carnitine transporter	AT1G73220	Organic cation/carnitine transporter 1	*OCT1*	-1.11	0.46	51.96
Polyol transporter	AT2G18480	Probable polyol transporter 3	*PLT3*	-1.18	0.44	137.94
Boron transporter	AT1G74810	Putative boron transporter 5	*BOR5*	-1.55	0.34	10.90
Ascorbate transporter	AT1G49960	Nucleobase-ascorbate transporter 4	*NAT4*	-1.65	0.32	229.76
Aminoacid transporter	AT4G35180	Lysine histidine transporter-like 7	*LHT7*	-1.79	0.29	73.27
Proline transporter	AT2G36590	Proline transporter 3	*PROT3*	-1.80	0.29	4.22
Potassium Transporter	AT3G56290	Potassium transporter	*-*	-1.86	0.28	14.91
	AT4G13420	Potassium transporter	*POT5*	-3.27	0.10	217.84
Aluminum transporter	AT1G08430	Aluminum-activated malate transporter 1	*ALMT1*	-2.50	0.18	548.13
Sulfate transporter	AT1G22150	Sulfate transporter 1.3	*SULTR1;3*	-4.46	0.05	2.23
Cyclic nucleotide-gated ion channel	AT3G17690	Putative cyclic nucleotide-gated ion channel 19	*CNGC19*	-1.37	0.39	209.32
	AT5G54250	Cyclic nucleotide-gated ion channel 4	*CNGC4*	-2.37	0.19	2.47
Aquaporin channel	AT2G39010	Probable aquaporin PIP2-6	*PIP2-6*	-2.23	0.21	39.41
	AT4G01470	Aquaporin TIP1-3	*TIP1-3*	-4.51	0.043	0

FC, Fold chamge; RT, Treated Root.

The reliability of transcriptome sequencing was confirmed using qRT-PCR, which investigated several DEGs, including those with suspected involvement in root-based carbon uptake and fixation (*NRT2.1*, *PEPC3*, *SULTR1;1*; *SULTR1;2*; *SLAH3*, *βCA4*) between control and treated roots, as well as between leaf samples. As shown in **Supplementary Figure 12**, the qRT-PCR results were largely consistent with the transcriptome data. Furthermore, linear regression analysis was conducted to assess the correlation between qRT-PCR and RNA-seq, yielding R^2^ values of 0.9564 for the roots and 0.9164 for the leaves, indicating a strong positive correlation between the two.

### 3.4 Physiological evaluation of possible inorganic transporters

After optimizing a growth-promoting treatment and proving the uptake and fixation of the added carbon, as well as identifying gene candidates that could take part in the uptake and fixation of the carbon, their gene knockout mutants were assessed under the growth-promoting system (2 mM NaHCO_3_ at pH 5.6) to validate their function.

The data analyzed for the screening of candidates to be evaluated were based on a *p-*value of 0.05 and a log_2_ FC of 0, genes with high expression levels, even if their FC did not change less than two-fold between control and treated plants, were studied based on literature information. DEG-lists are available as **Supplementary Tables 12** and **13**, our candidate list is as **Table 3**.

**Table 3:** Gene candidates from the transcriptome sequencing of *Arabidopsis thaliana* when treated with the growth promoting system (2mM NaHCO_3_) that might take part in root-based uptake or fixation of inorganic carbon.

Gene	Abbreviation	ID	Mutant studied	Expression level in RT	FC in RT vs. RC	Expression level in LT	FC LT vs. LC
Aquaporin	*AtPIP1;2, PIP1B*	AT2G45960	SALK_019794C	45901,93	1,33	24003,28	n.s.
	*AtPIP1;2, PIP1B*	AT2G45960	SALK_145347C	45901,93	1,33	24003,28	n.s.
	*AtPIP1;3, PIP1-3*	AT1G01620	SALK_051107C	35756,87	1,67	7174,58	n.s.
	*AtPIP2;6, PIP2-6*	AT2G39010	SALK_029718C	39,42	0,21	13582,52	1,45
PEP-carboxylase	*PEPC3*	AT3G14940	SALK_143289	6885,54	0,65	739,60	n.s.
Carbonic anhydrase	*βCA4*	AT1G70410	SALK_067006C	4270,04	0,75	6863,07	n.s.
Slow-type anion channel	*SLAH3*	AT5G24030	SALK_207089C	8093,20	2,67	349,78	n.s.
Sulfate transporter	*SULTR1;2*	AT1G78000	SALK_122974	18930,47	1,44	351,05	n.s.

Gene expression values and fold change (FC) of selected genes are shown in treated roots (MSRC vs. MSRT) and leaves (MSLC vs. MSLT). n.s., non-significant difference.

**Figure 6:**
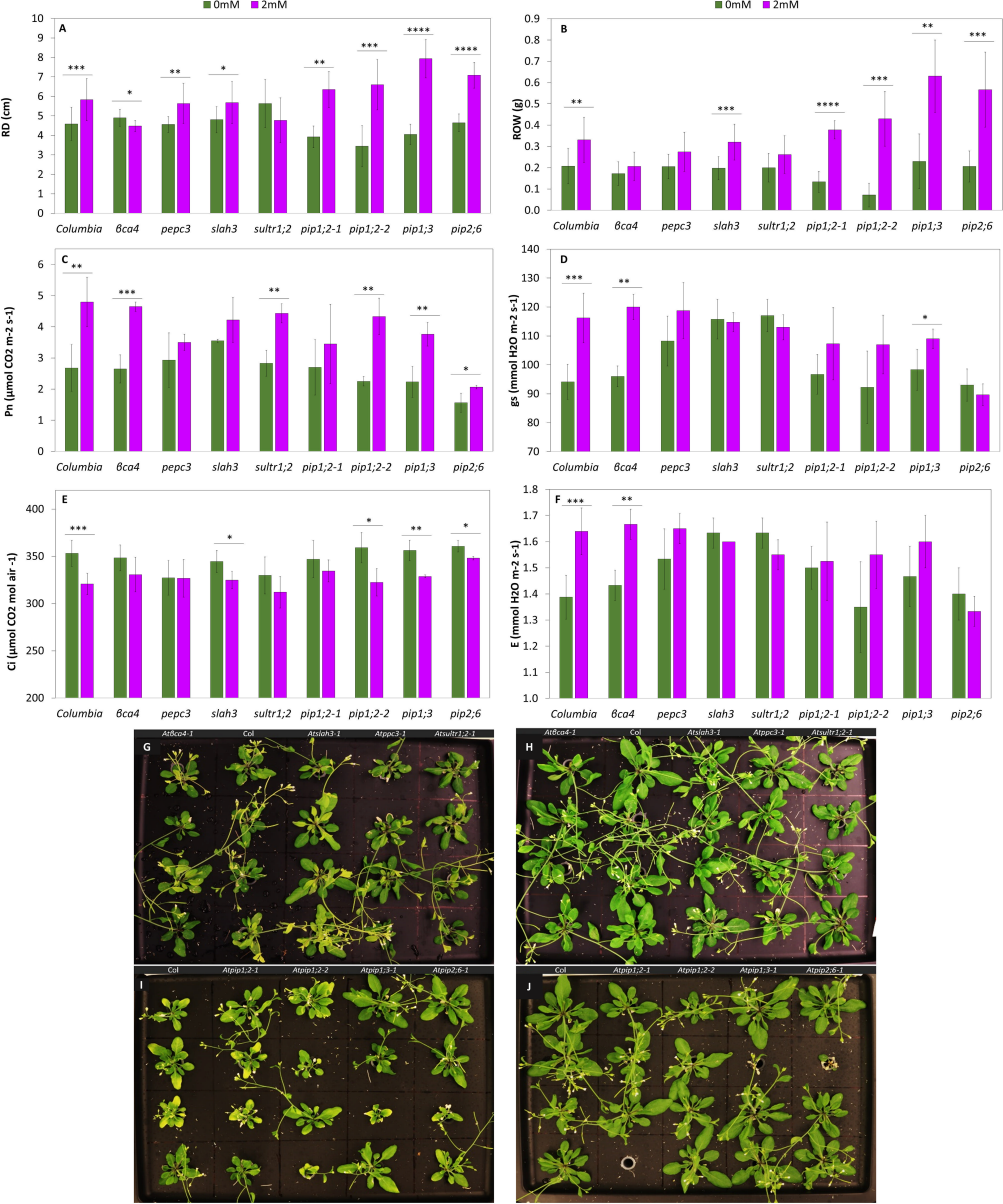
Growth parameters and gas exchange analysis of Col-0 and the mutants in the rosette. Rosette diameter **(A)**, Rosette weight **(B)**,. Net photosynthesis rate **(C)**, stomatal conductance **(D)**. Intracellular CO_2_ concentration **(E)**, Transpiration rate **(F)**, **(G)** Col-0 and mutants *(βca4, slah3, pepc3* and *sultr1;2*) with 0mM NaHCO_3_ pH 5.6. **(H)** Col-0 and mutants (*bca4, slah3, pepc3* and *sultr1;2*) with 2mM NaHCO_3_ pH 5.6. **(I)** Col-0 and mutants (*pip1;2-1, pip1;2-2, pip1;3*, and *pip2;6*) with 0mM NaHCO_3_ pH 5.6. **(J)** Col-0 and mutants *(pip1;2-1, pip1;2-2, pip1;3*, and *pip2;6*) with 2mM NaHCO_3_ pH 5.6. Each value represents the mean ± SD of n=12 for growth parameters and n= 4 for gas exchange parameters. Asterisks indicate statistically significant differences between the control and the treated plants (*p*-value ≤0.05*, ≤0.01 **, ≤0.001 ***, Student t-test). The scale bar is 5 cm.

**Figure 7:**
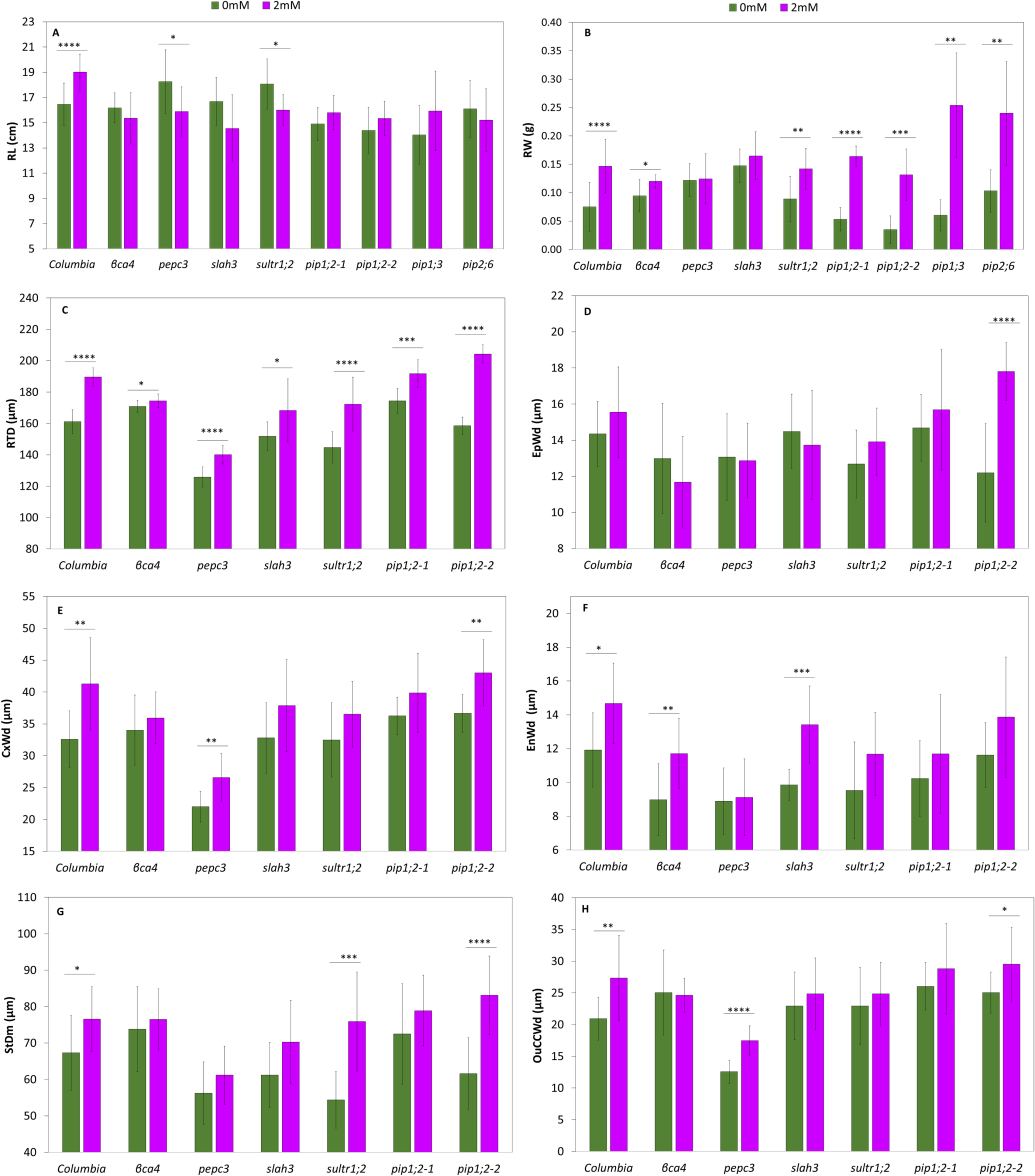
Growth parameters and root microscopy analysis of Col-0 and the mutants in the roots treated with 0mM NaHCO_3_ and 2mM NaHCO_3_. Root length **(A)**, root weight **(B)**, root diameter **(C)**, epidermis width **(D)**, Cortex width **(E)**. **(F)** Endodermis width **(F)**. Stele diameter **(G)**. Outer cortical cell width **(H)**. Each value represents the mean ± SD of n=12 for growth parameters and the root analysis. Asterisks indicate statistically significant differences between the control and the treated plants (*p*-value ≤0.05*, ≤0.01 **, ≤0.001 ***, Student t-test).

Growth parameters, photosynthetic parameters, and root microscopy results were evaluated for both the wild-type and mutant plants. All growth parameters including rosette diameter (ROD) (**Figure 6A**), rosette weight (ROW) (**Figure 6B**), root length (RTL) (**Figure 7A**), and root weight (RTW) (**Figure 7B**) were significantly increased in treated Col-0 plants compared to the control, whereas distinct trends were observed in the mutant lines.

Regarding the shoot system, the ROD of Col-0 exhibited a substantial growth of 27%. Mutants such as *pepc3* and aquaporin displayed a notable increase, with aquaporins nearly doubling the growth percentage by 50% between the control and the treatment. On the other hand, *βca4* showed a significant decrease in ROD, (approximately 9%). The *slah3* and *sultr1;2* mutants did not exhibit any significant differences as a result of the bicarbonate treatment (**Figure 6A**). The ROW of Col-0 demonstrated a substantial increase of 57%. The aquaporin mutants exhibited a significant increase of over 100%, particularly *pip1;2-2* with more than a 500% difference, and *slah3* also showed a significant increase of 60% (**Figure 6B**, **Supplementary Table 14**). Apart from comparing the treated plants in each genotype to their own control, it was also worth comparing the treated plants of different genotypes, here we could see that *pip1;3*, and *pip2;6* had a bigger ROD and ROW compared to treated Col-0.

The shoot system of the plants were not just compared by measuring growth parameters, photosynthetic analysis was also conducted. We measured gas exchange to better understand the impact of the 2 mM NaHCO_3_ treatment. Col-0 showed an increase in all the parameters assessed, except for the Ci, which decreased by 9% (**Figure 6E**). P_n_ was significantly increased in the treated *βca4, sultr1;2*, and aquaporin mutants, except for *pip1;2-1* (**Figure 6C**). While P_n_ in *sultr1;2* was significantly higher than the control, with a difference of 57%, the other evaluated parameters decreased compared with the control, but the difference was not statistically significant. Similar results were obtained for *slah3* and *pip2;6.* Notably, *βca4* was the only mutant that exhibited a significant increase in E and gs of 17% and 25%, respectively, similar to the wild-type (**Figures 6F, D**) (**Supplementary Table 15**). In a comparison of the mutants and the *Col-0* in the P_n_ values and transpiration rates of the *pip2;6* had the lowest values within all the mutants.*, gs.* Representative images of the controls (**Figures 6G, I**) and treated (**Figures 6H, J**) Col-0 and the mutant lines are provided.

In terms of root measurements, the wild-type exhibited a significant 15% increase in RTL compared to the control, while *pepc3*, and *sultr1;2* showed significant decreases of 13% and 11%, respectively (**Figure 7A**). Regarding RTW, Col-0 showed a significant increase of 88% with the treatment, except for *pepc3*, where all the mutants increased their RTW. The aquaporin mutants displayed a significant increase of over 140% (**Figure 7B**) (**Supplementary Table 14**). When comparing with Col-0 the RTW of *pip1;3* and the *pip2;*6 RTW was higher.

**Figure 8:**
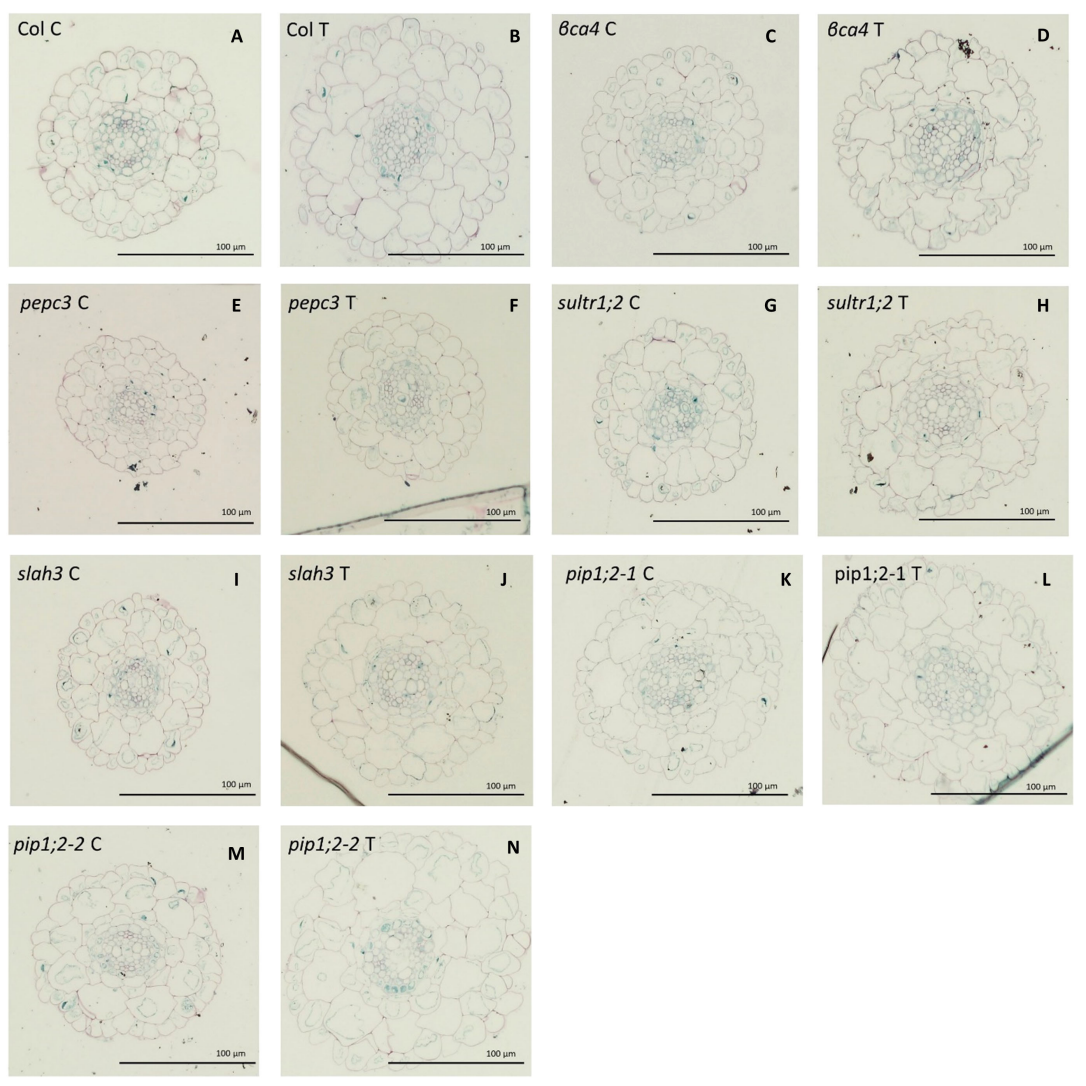
Root ultrastructure for the different for Col-0 and the mutants under 0 mM NaHCO_3_ and treated with 2mM NaHCO_3_ at pH 5,6. Col-0 control **(A)**, Col-0 treated **(B)**, *βca4* control **(C)**, *βca4* treated **(D)**, *pepc3* control **(E)**, *pepc* treated **(F)**, *sultr1;2* control **(G)**, *sultr1;2* treated **(H)**, *slah3* control **(I)**, *shla3* treated **(J)**, *pip1;2-1* control **(K)**, *pip1;2-1* treated **(L)**, *pip1;2-2* control **(M)**, *pip1;2-2* treated **(N)**. The scale bar is for 100 µm.

In the above experiments, we studied mutants of genes with high expression levels in the roots and with suspected involvement in carbon uptake (aquaporins, *sultr1;2*), fixation (*βca4, pepc3*), and toleration of bicarbonate treatment, as well as their role in plant homeostasis (*slah3*). The leaf-specific aquaporin gene *pip2;*6 was also studied for comparison. The considerably inferior growth and photosynthetic parameters of a given mutant at 2 mM pH 5.6 NaHCO_3_ compared to Col-0 could indicate a defective carbon uptake or fixation capacity, and thus, the role of the mutated gene in the process. As mentioned above, many of our mutants (*sultr1;2*, *slah3, βca4*, and *pepc3*) did not show a characteristic increase in ROW and RTW or photosynthetic activity as a result of bicarbonate treatment, as was expected. However, to our surprise, the *pip1;3* and the *pip2;*6 aquaporin mutants were rather superior to Col-0 in many parameters, especially growth indicators, showing that these genes might have a negative role in the carbon uptake. Previous research e.g (Rogers et al., 1992) has shown that inorganic carbon treatment results in increased root growth and is associated with the root ultrastructure involved in carbon uptake. We validated this hypothesis and compared the growth of Col-0 and the KO mutants (**Figures 7**, **8**; **Supplementary Table 16**).

Root diameter (RTD) significantly increased in Col-0 and all mutants, except for *βca4*, by more than 10%. The *pepc3* mutant had the thinnest roots, whereas the *βca4* mutant showed the smallest increase as a result of the treatment, as previously mentioned (**Figure 7C**). In general, the bicarbonate treatment slightly increased the epidermis width (EpWd) in Col and the mutants, with the exception of *pip1;2-2*, which exhibited a greater increase of approximately 46%. In the case of *βca4, pepc3*, and *slah3* it decreased, but the difference was not statistically significant (**Figure 7D**). The endodermis width (EnWd) showed significant positive differences between the control and treated plants for Col-0 *βca4*, and *slah3*, with values higher than 20%. No significant differences were between the control and the treated *pepc3* and aquaporin mutants (**Figure 7F**). The increase in the width of the cortex (CxWd) (**Figure 7E**) in Col-0 between the control and treatment was significant (27%). Similarly, the mutant genes *pepc3* and *pip1;2-2* showed a significant increase between the control and treated plants, approximately by 20%. However, no significant differences were observed among *βca4, sultr1;2, pip1;2-1*, and *slah3* mutants (**Figure 7F**). The stele diameter (StDm) increased significantly in Col-0 by 14%, whereas the *sultr1;2* mutant showed a substantial increase of 39%, and the *pip1;2-2* mutant showed an increase of 35%. However, no significant differences were observed between the other mutants and the control and treated plants (**Figure 7G**). About the outer cortical cell width (OuCCWd), Col-0 showed a significant increase of 31%) between the control and the treated plants. The *pepc3* and *pip1;2-2* mutants significantly increased by 39% and 18%, respectively. The *βca4, pip1;2-1, slah3*, and *sultr1.2* mutants did not show significant differences (**Figure 7H**). Therefore, in general, many of our mutants (especially *βca4, sultr1;2, slah3*) showed some deviations from Col-0 in the root ultrastructure caused by bicarbonate treatment (especially in StDm, CxWd, and OuCCW), indicating that the mutated genes were missing for the uptake/fixation/toleration of bicarbonate.

## 4 Discussion

Theoretically, root-based inorganic carbon uptake could boost photosynthetic activity by improving carbon supply. In this study, we characterized the physiology and mechanism of root-based inorganic carbon uptake by *Arabidopsis thaliana* (Col-0). First, a hydroponics-based inorganic carbon treatment was optimized, which served as the basis for further experiments.

Inorganic carbon uptake and fixation from solutions has been proved in beans and sunflowers only at low pH (mostly in carbon dioxide form) (Shimono et al., 2019). Arabidopsis is capable of root-based inorganic carbon uptake at a low (5.9) (Pérez-Martín et al., 2021) and higher pH (pH 7, 8.3) (Dąbrowska-Bronk et al., 2016; Pérez-Martín et al., 2021). However, a higher pH might decreases the availability of some macronutrients such as phosphorus, calcium, and magnesium, and considerably hampers the uptake of micronutrients such as zinc, copper, manganese, and especially iron (Fimbres-Acedo et al., 2023). In our study, the growth promotion was achieved by supplying 2 mM NaHCO_3_ at a low pH (5.6). Nutrient deficiency could explain the lack of growth promotion in the presence of MS medium and 2 mM NaHCO_3_ at high pH (7.2); however, we do not exclude the possibility that high-pH growth promotion could work with different media. Another fact also argues that bicarbonate uptake could be more effective at low pH, plant roots tend to take up anions with proton co-transporters and so there is a proton requirement, though part of bicarbonate could turn into CO_2_ at low pH which decreases the efficiency of the mechanism. Our candidates for bicarbonate uptake are SULTR and NRT pumps which are also known to be proton-coupled symporters (Sun and Zheng, 2015; Wang et al., 2021); and literature evidence also shows higher sulfate ion uptake with SULTRs at lower pH (Cao et al., 2013; Wang et al., 2021).

As part of the growth promotion, the root ultrastructure was also studied, as earlier reports showed root growth, thickening of the cortex, and the role of root hairs in bicarbonate uptake (Rogers et al., 1992; Dąbrowska-Bronk et al., 2016; Wang et al., 2020). Our results confirmed previous reports that the root cortex widens in response to elevated carbon supply, however, we did not observe a significant change in root hairiness. However, there was an increase in the number of lateral root branches, which could reflect a higher demand for plant growth and mineral uptake.

The beneficial bicarbonate treatment used in this study (2 mM NaHCO_3_ at pH 5.6) not only improved the growth parameters but also the photosynthetic ones. In Col-0, P_n_ was higher as a result of the carbon treatment, similar to that reported by Wang et al. (2020); however, in our study, the transpiration rate and stomatal conductance increased but decreased in Wang et al. (2020). Notably, Wang et al. (2020) used 720 ppm CO_2_ different from ours since we used a soluble form of inorganic carbon added to the roots. Li et al. (2023) treated *Coix lacryma-jo*bi with different concentrations of NaHCO_3_. Their 2 mM NaHCO_3_ treatment increased the plant biomass, P_n_, gs, and E, similarly to our results.

Although we successfully established a hydroponics-based growth-promoting inorganic carbon treatment, it was essential to conclusively prove that the plants took up and fixed the supplied carbon and, if possible, the ratio and thus the importance of this carbon. Carbon uptake and fixation have been studied using isotope labeling and fluxomic measurements. A higher level of ^13^C aspartic acid in the xylem sap than in the phloem can be a result of root-based PEPC-based fixation. PEPC is known to be less discriminative against the ^13^C isotope compared to the green-tissue specific RuBisCo (Tcherkez et al., 2011). Miszalski et al. (2023) reported lower discrimination for ^13^C in roots compared to the lamina or leaf bundle of *Plantago lanceolata* L. In our experiments, the concentration and ratio of ^13^C-aspartic acid increased slightly when the roots were provided with ^13^C. Fixation of bicarbonate by PEPC in roots has also been demonstrated by others by detecting increased oxaloacetate-derived carbonic acid (malate and citrate) and amino acid (aspartate, asparagine, lysine, methionine, threonine, and isoleucine) in the xylem sap of bicarbonate-treated roots (Bialczyk and Lechowski, 1992, 1995; Yang et al., 1994; Alhendawi et al., 1997; Wanek and Popp, 2000; Nomura et al., 2006). Hibberd and Quick (2002) found evidence of C_4_-like assimilation of root-derived carbon in the stems of tobacco plants. However, the slight increase in the labeled product in our study and the decrease in *PEPC* gene expression (see above) led us to conclude that PEPC-based fixation in the roots could not have been the main fixation pathway of hydroponically added carbon in *A. thaliana*, and it would have placed huge energy demand on the roots.

On the other hand, the concentration and ratio of ^13^C-labeled sucrose greatly increased in the phloem sap as a result of the treatment in our work, so the transport of inorganic carbon to the shoots and Calvin-cycle-based fixation might have been of pivotal importance. Higher stomatal conductance of the treated plants could have also influenced the results as it leads to higher diffusion of ^13^CO_2_, less discrimination against ^13^C, and its fixation from the air through stomata (Śliwa et al., 2019; Miszalski et al., 2023). Re-fixation of carbon originally fixed in the roots into organic molecules cannot be excluded either. Therefore, it is difficult to determine the contribution of this root-supplied inorganic carbon to the production of photoassimilates. The percentage of ^13^C-labeled sucrose grew by 12-fold and reached approximately 2% of all sucrose; however, the contribution of hydroponically added ^12^C could have been higher because ^13^C CO_2_ is known to diffuse slower and is discriminated against RuBisCo and other Calvin-cycle enzymes (Tcherkez et al., 2011).

Transcriptome sequencing was used to identify different pathways that show upregulation and downregulation during bicarbonate treatment. The expression of many more genes changed significantly in the roots (1472 DEGs) than in the leaves (341 DEGs), this may indicate the prevalence of root-based factors of inorganic carbon uptake when a source of carbon is added directly to the roots. Notably, some pathways were upregulated, as observed in earlier studies that utilized high-concentration inhibitory bicarbonate treatments. For instance, iron uptake mechanisms were activated; in fact, the ferric reduction oxidase 5 gene (AT5G23990) showed the highest fold change (FC) of all DEGs (more than 3000-fold) and the GO term “iron ion transmembrane transporter activity” and “iron ion binding” were upregulated in the roots, but “iron ion homeostasis” decreased in the leaves. High-concentration bicarbonate treatment is known to cause iron deficiency (Lucena, 2000) and upregulate iron-uptake mechanisms (Msilini et al., 2009; Pérez-Martín et al., 2021). Our plants might have struggled to take up iron, but with this 2 mM NaHCO_3_ treatment, they were successful; they were healthy and larger than the controls. Other than *FRO5*, another bicarbonate-protective gene, *SLAH3* (AT5G24030), was also induced in our plants. Slow-type anion channels are efflux-type nitrate and chloride channels, carbon sensors in stomata, and are involved in their closure as a result of high inorganic carbon levels (Li et al., 2022). However, *SLAH3* is also highly expressed in the root xylem pericycle and pumps nitrate into the xylem, which has been shown to be a protective mechanism against high bicarbonate-induced toxicity, given the signaling role of nitrate (Duan et al., 2018). The *SLAH3* mutants in our bicarbonate treatments showed somewhat reduced growth and different root structures compared with the wild type.

The low concentration of bicarbonate applied in this study and other studies may have caused beneficial and stimulating stress. In our work, carbon fixation was proven by fluxomics, and literature also suggests increased N acquisition like in tomato (Navarro et al., 2000), poplar and elder (Wanek and Popp, 2000) therefore, we believe this eustress is only partly responsible for the observed beneficial growth-promoting effect of bicarbonate treatment. The upregulation of the GO term “response to nitrogen compound” was observed in both the roots and leaves of the plant. This upregulation occurs when plants experience high levels of CO_2_, leading to an imbalance between carbon and nitrogen. Plant productivity relies on its ability to take up nitrogen and coordinate the stimulation of carbohydrate production and growth by enhancing photosynthetic processes (Li et al., 2017). The upregulation of nitrate uptake and reduction is influenced by glucose and sucrose, which increases the expression of genes involved in nitrate transport (*NRT*) and nitrate/nitrite reduction (*NIA/NIR*) families (Thompson et al., 2017).

Since the photosynthesis, N-assimilation, and S-assimilation pathways are co-regulated, changes in one of them can influence the others (Lee and Kang, 2005). At higher CO_2_ levels, upregulation of sulfate uptake and reduction was expected as happened in our study where the GO term “sulfur compound metabolic process” was downregulated but the KEGG term “cysteine and methionine metabolism” was upregulated. This predicted upregulation is a result of increased protein synthesis due to higher CO_2_ levels, which leads to a greater demand for sulfur-containing amino acids such as cysteine and methionine. Cysteine is not only an important building block for proteins but can also be used to produce methionine. Methionine, similar to cysteine, is important for protein synthesis and playing a role in initiating mRNA translation. Additionally, it serves as a precursor for S-adenosyl methionine (SAM), which regulates crucial cellular processes such as cell wall biosynthesis and cell division (Hacham et al., 2013).

GO terms related to homeostasis in response to high levels of inorganic carbon in the roots and leaves suggest that the addition of bicarbonate to the root environment could have affected internal pH and nutrient availability, potentially causing osmotic, ionic, and oxidative stress (Li and Yang, 2023). To sustain plant growth, ion and pH homeostasis must be maintained. The upregulation of GO terms, such as the ‘response to oxidative stress’, ‘cation and ion homeostasis’, ‘cellular homeostasis’, and ‘chemical homeostasis’ in the roots, reflects the presence of these stresses. Cellular pH is tightly regulated to ensure optimal conditions for biochemical reactions, and plant cells possess numerous transport systems comprising cation/H^+^ and anion/H^+^ exchangers that maintain pH homeostasis in different subcellular compartments as well as transmembrane H^+^ gradients as a proton motive energy source (Rottet et al., 2021).

The GO term ‘trehalose biosynthetic process’ was upregulated both in the roots and leaves. The trehalose-6-phosphate (T6P) signaling pathway regulates the relationship between sources and sinks under different environmental conditions. This regulation leads to an increase in soluble saccharide content, resulting in a significant increase in aboveground weight, plant height, and stem diameter of the plant (Li et al., 2024). T6P has emerged as an important signaling metabolite that regulates carbon assimilation and sugar status in plants, plays an essential role in plant development, and is an important structural component of plant cells (Ponnu et al., 2011). Changes in photosynthetic capacity in plants with altered T6P levels are thought to be influenced by T6P’s impact on leaf development, particularly on cell division and cell wall biosynthesis. In addition to its role in regulating carbohydrate metabolism, T6P is also important for normal plant development (Ponnu et al., 2011). T6P is also believed to play a role in upregulating multiple macronutrient pathways when carbon levels are high, as demonstrated by Wingler et al. (2012).

Interestingly, the expression of high-level β-carbonic anhydrases (*βCA1* and *βCA4*) did not increase as a result of bicarbonate treatment in the transcriptomic analysis. The level of *βCA4* significantly decreased in the roots, and the expression was higher in the leaves. This contrasts with the results of Dąbrowska-Bronk et al. (2016), who found increased expression of many *βCA* genes, especially *βCA1* and *βCA4*, following bicarbonate treatment, although the experimental conditions were different from our research (hydroponically grown plants at pH 5.6 vs. irrigating potted plants with a pH 7 solution). However, the *βca4* mutants clearly showed growth differences from the wild type in our bicarbonate treatments; therefore, we agree with the authors that “CAs allow to use of soil HCO_3_^−^ ions as a substrate for photosynthesis in foliar tissues” (Dąbrowska-Bronk et al., 2016).

From the various mechanisms, that we hypothesized would participate in root-based carbon uptake, many aquaporin channel genes were activated as a result of the treatment. Some aquaporin genes, such as the well-characterized carbon channel *AtPIP1;2* (Heckwolf et al., 2011) excelled with very high expression levels but were only slightly induced further because of the treatment (FC = 1.33). On the other hand, many nitrate, phosphate, and sulfate anion channels, both high- and middle-level channels (*e.g.*, *SULTR1;1*, *SLAH3*, *NRT2.1, and PHO1*) were significantly induced (FC of 2–11). It has been hypothesized that some anion transporters may also have some affinity for bicarbonate [reviewed by Poschenrieder et al., 2018]. Therefore, increased expression of these anion transporters might take up bicarbonate ions, or they might be required for the uptake of other nutrients to sustain increased growth. As was already mentioned, we hypothesize that *SULTR*s and *NRT*s take up bicarbonate ions into the root, while PHO1 as an efflux-type phosphate channel pumps, phosphate into the xylem (Stefanovic et al., 2011) and it might also pump bicarbonate.

Among anion channels, sulfate transporters have been implicated in having some affinity for bicarbonate (Frachisse et al., 1999; Poschenrieder et al., 2018; Wang et al., 2021). Arabidopsis has two high-affinity sulfate transporters (*SULTR1;1* and *SULTR1;2*) that represent the sulfate uptake activity at the root surface (Maruyama-Nakashita et al., 2003). *SULTR1;2* is predominantly expressed in the root cortex, involved in importing sulfate from the environment into the root, and is not a very specific transporter; it transports selenate and chromate as well (Shibagaki et al., 2002; Maruyama-Nakashita et al., 2003; Xu et al., 2021). In our experiments, the high expression level of the sulfate transporter *SULTR1;2* was slightly further induced (FC = 1.44), while the lower level *SULTR1;1* was greatly induced (FC = 3.57) as a result of bicarbonate treatment. Under normal conditions with adequate sulfate in the soil, *SULTR1;2* is the primary transporter for sulfate uptake. However, under stressful conditions, *SULTR1;1* is the primary sulfate uptake transporter (Yoshimoto et al., 2002).

To determine whether aquaporin channels or anion pumps are more likely to be responsible for inorganic carbon uptake by roots, and the role of βCA and PEPC, gene-knockout mutants were tested for growth promotion under bicarbonate treatment. The *sultr1;2* mutant showed the greatest deviation (inferior growth promotion and photosynthetic activity) compared to the treated wild-type, which underlines the importance of this gene as a possible bicarbonate uptake pump. Treated roots exhibited various ultrastructural changes. While the growth and photosynthetic activity of many mutants were inferior to those of Col-0, the aquaporin mutant *pip1;3* showed superior performance; therefore, we believe that this gene could play a negative role in root-based carbon uptake. Based on our findings (transcriptome and mutants), we hypothesize that despite the low pH (carbon-dioxide-rich environment), Arabidopsis roots still preferentially take up bicarbonate ions with anion pumps, with possible candidates being *SULTR1;1*, *SULTR1;2* and *NRT2;1*, while *PHO1* might transport the bicarbonate into the xylem. The bicarbonate ion predominantly stays in this form in the root symplast, as the pH is usually slightly alkaline in the cytosol of plant cells, ranging from 7.2 to 7.5 (Felle, 2001). Carbon dioxide-permeable aquaporins may have a negative effect by venting the carbon dioxide out of the plant cells, produced by carbonic anhydrases. We found that β*CA4* expression decreased in the roots, possibly preventing the loss of CO_2_. The growth and photosynthetic activity of the *pepc3* mutants could not be boosted as much by our bicarbonate treatments as in the case of the Col-0. As it was mentioned, the production of labelled aspartic acid only slightly increased in the xylem sap of ^13^C-treated plants. Large-scale production of malate, another possible derivative of PEPC-based oxaloacetate, is yet more unlikely in the roots due to the requirement of reductive power (one NADPH corresponds to the energy of three ATPs), although the production of small amounts of malate and other organic acids has been detected in carbon-treated roots (Cramer et al., 1993; Alhendawi et al., 1997). However, PEPC-based carbon fixation can play a role in N acquisition; some articles refer to better ammonia assimilation in bicarbonate treated-roots (Marín-Peña et al., 2024), and others to nitrate, (Viktor and Cramer, 2005) in the roots, or both nitrate and ammonia (Andrews et al., 2019). Apart from the proposed bicarbonate uptake mechanism through proton-anion symporters, it can also be anticipated that CO_2_ (in this form, as the apoplastic pH is acidic in general: Felle, 2001) leaks into the xylem at root tips and branching sites where the Casparian strip has not formed. The xylem sap pH is generally acidic in Arabidopsis (Grunwald et al., 2021), therefore presumably mostly CO_2_ is transported up to the shoots in the xylem sap, enters the mesophyll cells, where it might be converted to bicarbonate at the alkaline cytoplastic pH but converted back to CO_2_ by CAs for fixation for RuBisCo.

**Figure 9:**
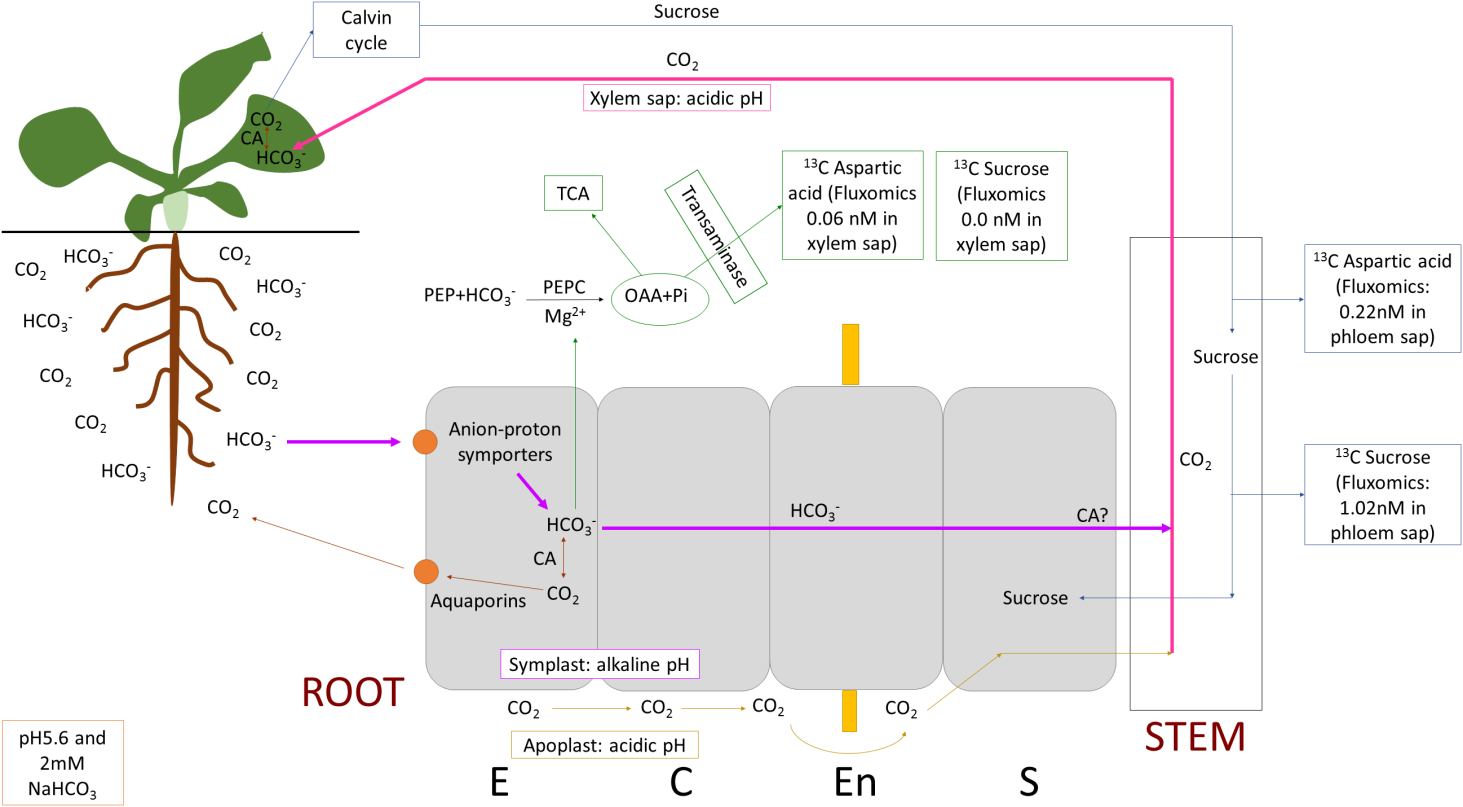
Proposed root-based inorganic carbon uptake and fixation mechanisms of *Arabidopsis thaliana*. Bicarbonate (HCO_3_^-^) ion is taken up by anion-proton symporters, a minority of bicarbonate is fixed in the roots by PEPC (phosphoenulpyruvate carboxylase), but the majority is transported up to the shoots by the xylem and fixed there in the Calvin-cycle after conversion to CO_2_ by carbonic anhydrases (CAs). Part of bicarbonate may be converted to CO_2_ by CAs and vented out of the root cells through aquaporin channels. However CO_2_ may leak into the xylem at root tips and branching sites where the Casparian strip has not formed. Note the pH of the different spaces (root/leaf symplast: alkaline, apoplast: acidic, xylem sap: acidic) which also affects carbon fixation and transport, inorganic carbon can be found predominantly as CO_2_ at lower and bicarbonate at higher pH but the conversion is slow without CAs. OAA stands for oxaloacetate, TCA, tricarboxylic acid cycle; E, epidermis; C, cortex; En, endodermis and S, stele.

Our study found growth-promoting effects of low-concentration bicarbonate treatment in Arabidopsis with root-based uptake. Higher growth was possibly regulated by trehalose-6-phosphate signaling, and various mechanisms have been proposed to support the increased growth, including anatomical changes (wider root cortex) and higher nitrogen and sulfur assimilation. Transcriptomic data revealed the involvement of various transmembrane transporter activities, cation and anion transport, and maintenance of homeostatic balance. Increased iron uptake and higher *SLAH3* expression alleviated the toxic effects of bicarbonate. In addition, we identified potential inorganic carbon transporters in the roots that can take up either HCO_3_^-^. Further studies are necessary to confirm these findings. Our investigation also revealed that unlike in leaves, aquaporins, especially *PIP1;3* may have a negative impact by venting carbon dioxide out of the plant in the roots. These results suggest that a minority of bicarbonate was fixed in the roots by PEPC, but the majority was transported up to the shoots by the xylem and fixed there by RuBisCo after conversion to CO_2_ by carbonic anhydrases. The proposed root-based inorganic carbon uptake and fixation mechanisms of *Arabidopsis thaliana* are presented on **Figure 9**. The study shed new light on the mechanisms of root-based inorganic carbon uptake, which could be relevant for boosting photosynthesis with carbon supply.

## Data Availability

The RNA-Seq data underlying this article are available in the National Center for Biotechnology Information Sequence Read Archive (NCBI SRA) at https://www.ncbi.nlm.nih.gov/sra/PRJNA1085068.
