# Supplementary material for: Root-based inorganic carbon uptake increases the growth of Arabidopsis thaliana and changes transporter expression and nitrogen and sulfur metabolism
Source: Front Plant Sci. 2024 Sep 6;15:1448432. doi: 10.3389/fpls.2024.1448432 (PMC11412874; doi:10.3389/fpls.2024.1448432)
Supplement: Supplementary file 1 [file DataSheet1.pdf]

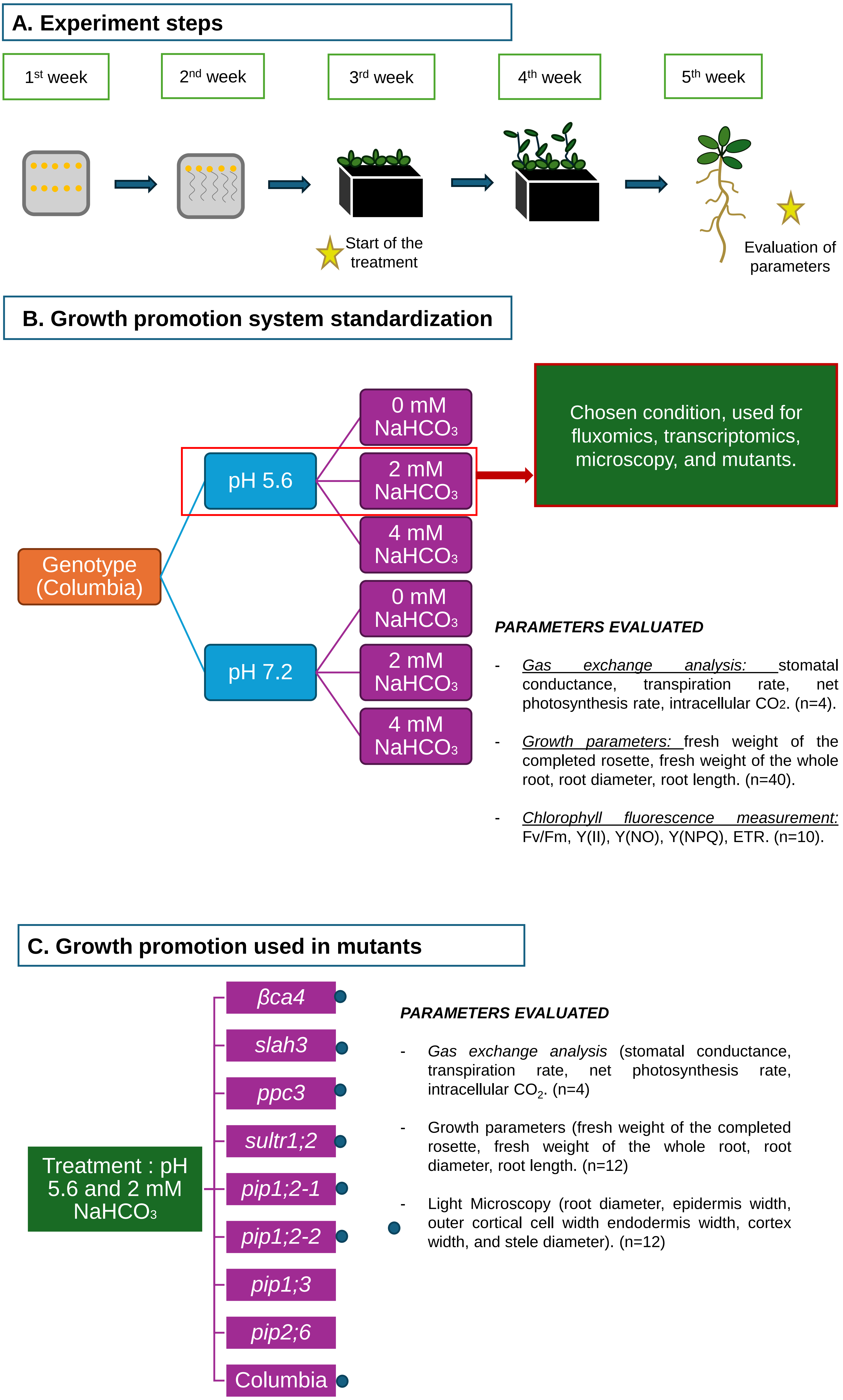

Supplementary figure 1. Diagram explaining the methodology used in this article. A. Growing of the hydroponic cultures. B. Growth promotion standardization experiment and evaluation. C. Growth promotion applied to the mutants selected to study.
