# Supplementary material for: Root-based inorganic carbon uptake increases the growth of Arabidopsis thaliana and changes transporter expression and nitrogen and sulfur metabolism
Source: Front Plant Sci. 2024 Sep 6;15:1448432. doi: 10.3389/fpls.2024.1448432 (PMC11412874; doi:10.3389/fpls.2024.1448432)
Supplement: Supplementary file 2 [file DataSheet2.pdf]

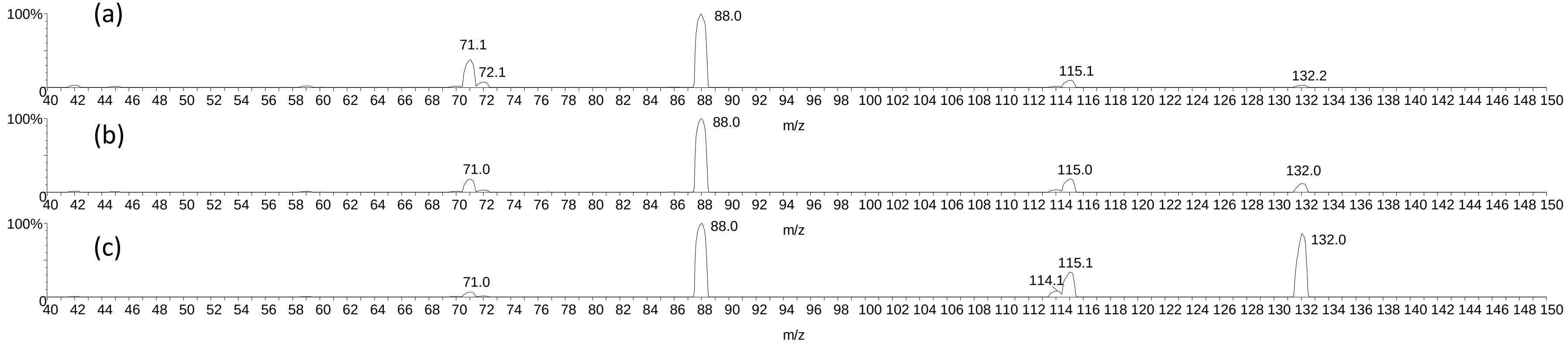

Supplementary Figure. S2 a, b, c: ESI-MS/MS fragments of the natural aspartic acid standard in the ESI negative mode on the 'A' (monoisotopic) isotopologue, *m/z* 132, at the collision energy settings of 20 eV, 15 eV and 10 eV, respectively.

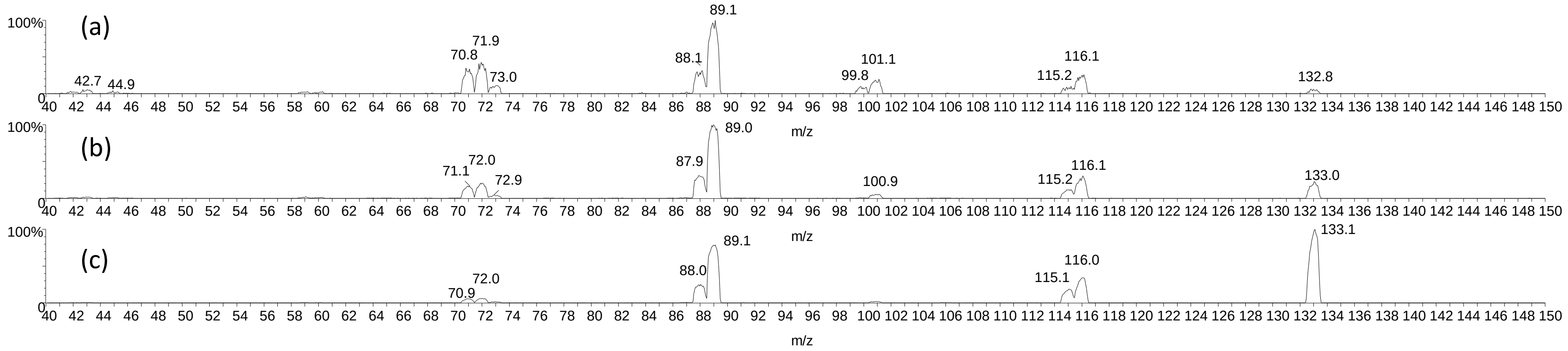

Supplementary Figure S3 a, b, c: ESI-MS/MS fragments of the natural aspartic acid standard in the ESI negative mode on the 'A+1' isotopologue, *m/z* 133, at the collision energy settings of 20 eV, 15 eV and 10 eV, respectively.

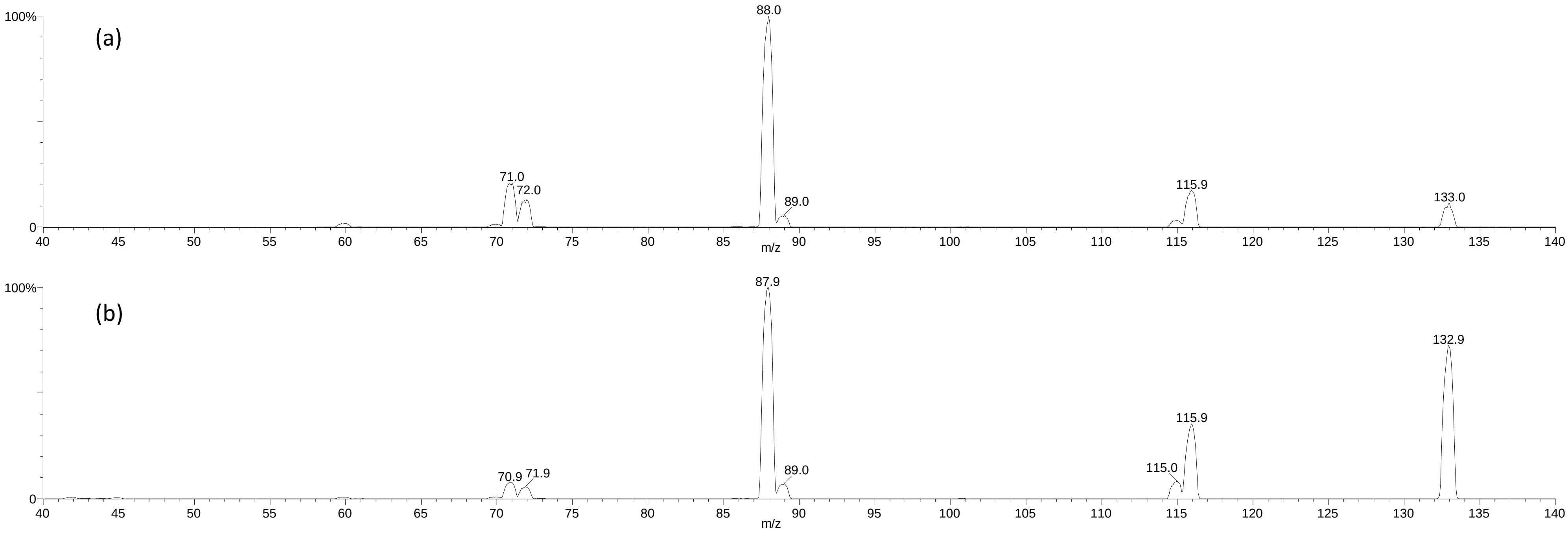

Supplementary Figure S4 a, b: ESI-MS/MS fragments of the DL-aspartic acid-4-^{13}C standard in the ESI negative mode on the 'A' (monoisotopic) isotopologue, *m/z* 133, at the collision energy settings of 18 eV and 12 eV, respectively.

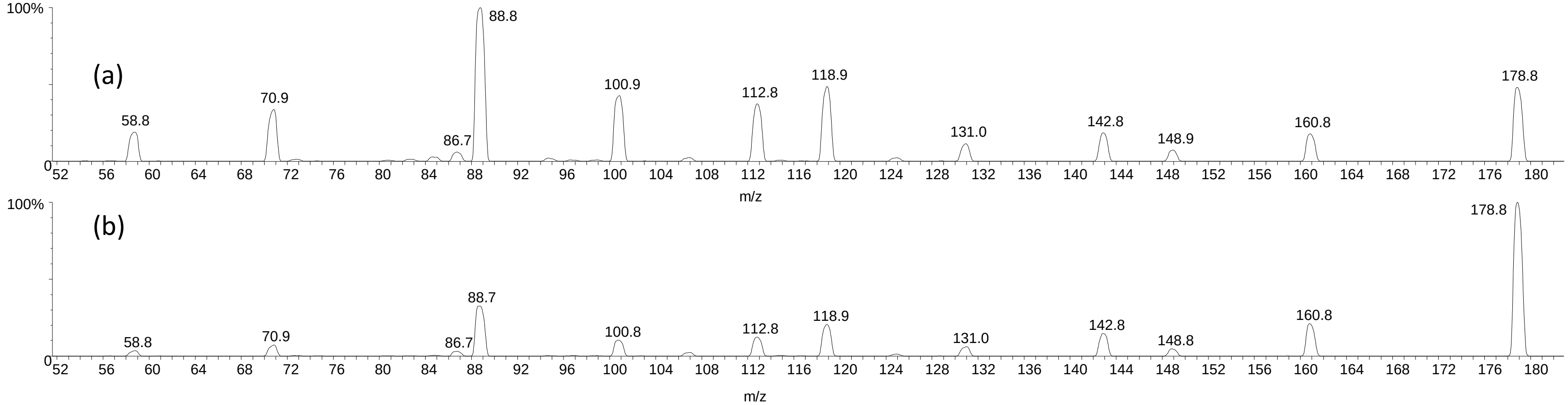

Supplementary Figure S5 a, b: ESI-MS/MS fragments of the natural sucrose standard in the ESI negative mode on the ’A’ (monoisotopic) isotopologue, *m/z* 341, at the collision energy settings of 18 eV and 12 eV, respectively.

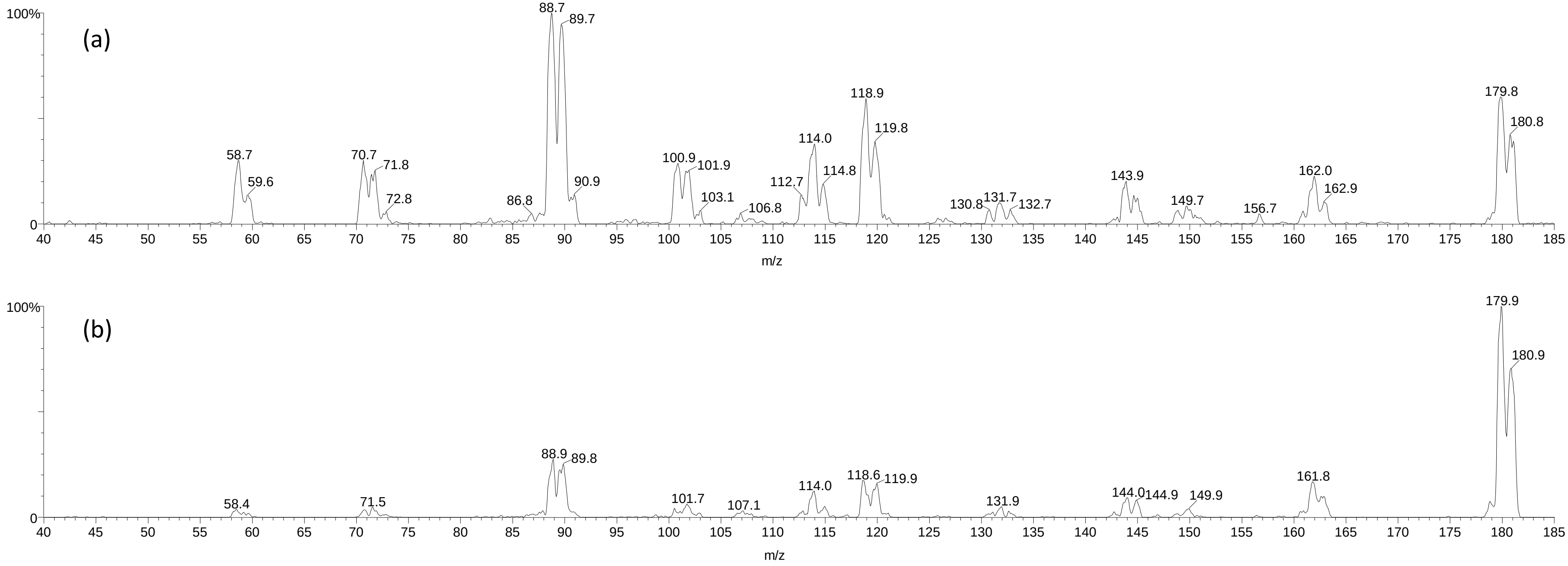

Supplementary Figure. S6 a, b: ESI-MS/MS fragments of the [1-^{13}Cglc]sucrose standard in the ESI negative mode on the ’A+1’ isotopologue, *m/z* 343, at the collision energy settings of 18 eV and 12 eV, respectively.
