# Supplementary material for: Root-based inorganic carbon uptake increases the growth of Arabidopsis thaliana and changes transporter expression and nitrogen and sulfur metabolism
Source: Front Plant Sci. 2024 Sep 6;15:1448432. doi: 10.3389/fpls.2024.1448432 (PMC11412874; doi:10.3389/fpls.2024.1448432)
Supplement: Supplementary file 3 [file DataSheet3.pdf]

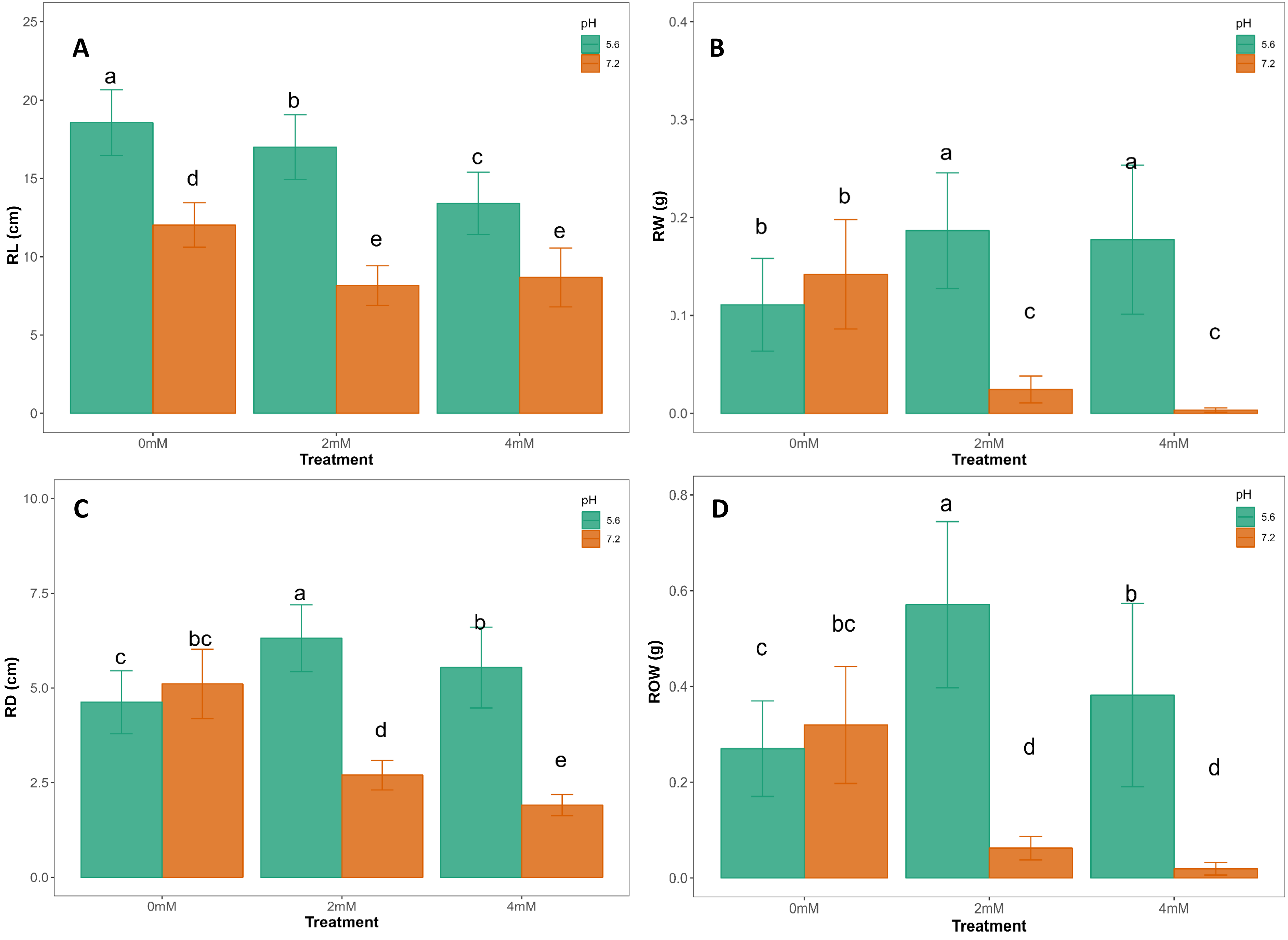

Supplementary Figure S7. Fresh weight of *A. thaliana* (Col-0) comparing three different concentrations of $NaHCO_3$ (0,2 and 4 mM) and two different pH (5.6 and 7.2). A. Root length. B. Root weight. C Rosette diameter. D. Rosette weight. Each value represents the mean ± SD of n=10. The letters represent the significant differences between the treatments P < 0.05 Tukey's HSD *post-hoc* test.

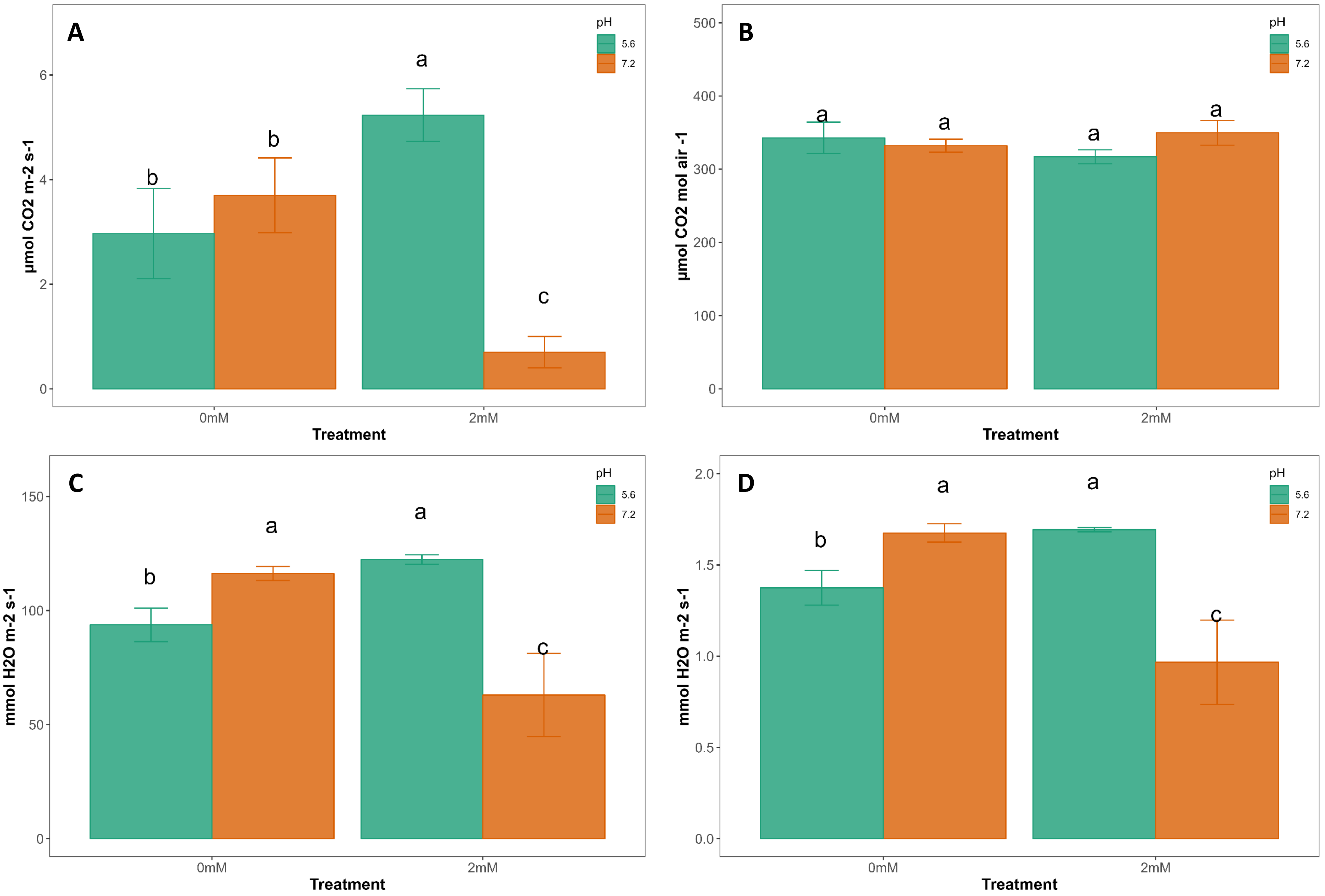

Supplementary Figure S8. Gas exchange analysis in *A. thaliana* (Col-0) comparing three different concentrations of NaHCO3 (0, 2 mM) and two different pH (5.6 and 7.2). A. Net photosynthesis rate. B. Intracellular CO_2. C Stomatal conductance. D. Transpiration rate. Each value represents the mean ± SD of n=4. The letters represent the significant differences between the treatments P < 0.05 Tukey's HSD *post-hoc* test. The 4mM treatment is not due to the inhibitory effects of the treatment.

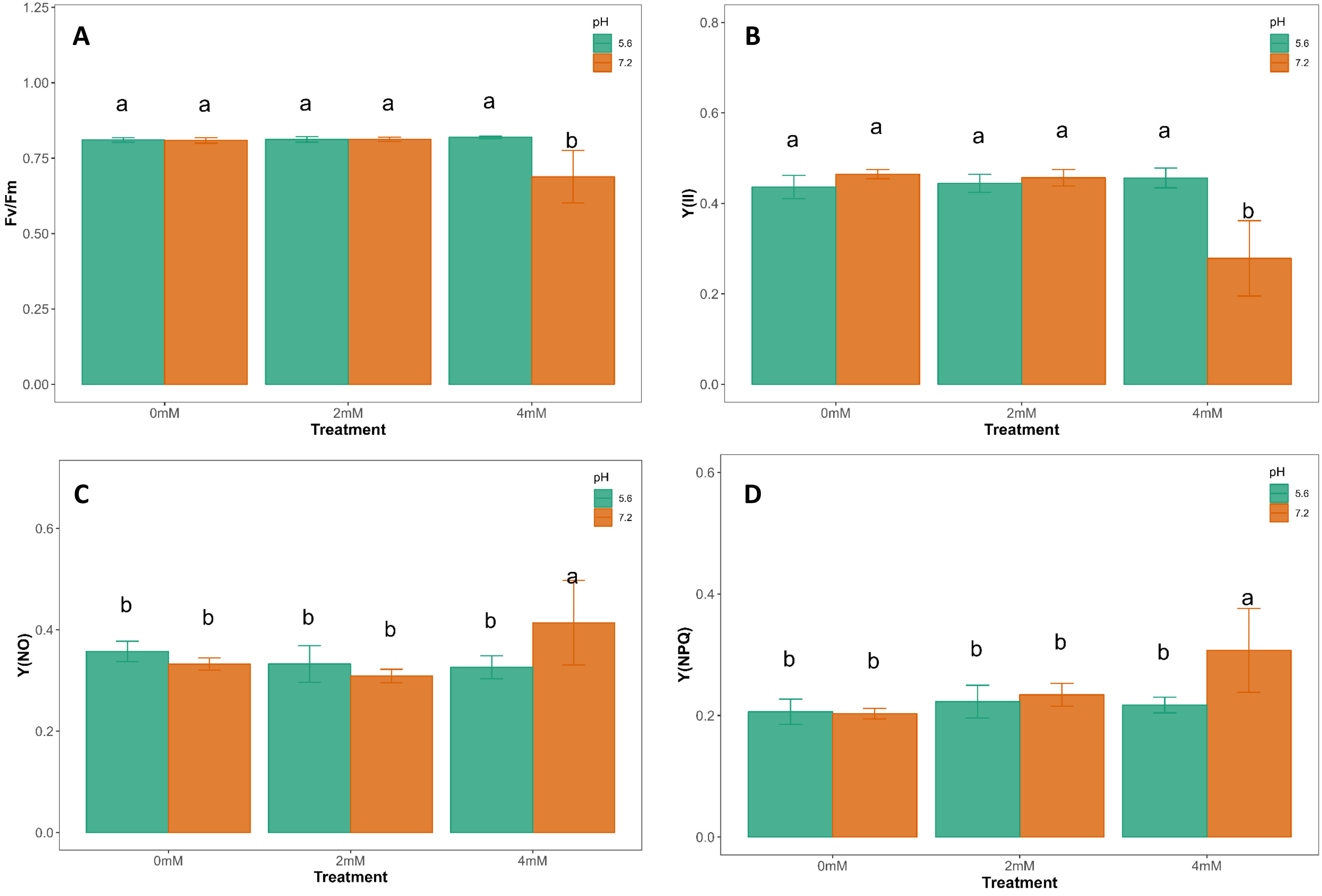

Supplementary Figure S9. Chlorophyll fluorescence imaging in *A. thaliana* comparing three different concentrations of NaHCO3 (0,2, and 4 mM) and two different pH (5.6 and 7.2). A. Fv/Fm. B. Y(II). C Y(NO). D. Y(NPQ). Each value represents the mean ± SD of n=10. The letters represent the significant differences between the treatments P < 0.05 Tukey's HSD *post-hoc* test.

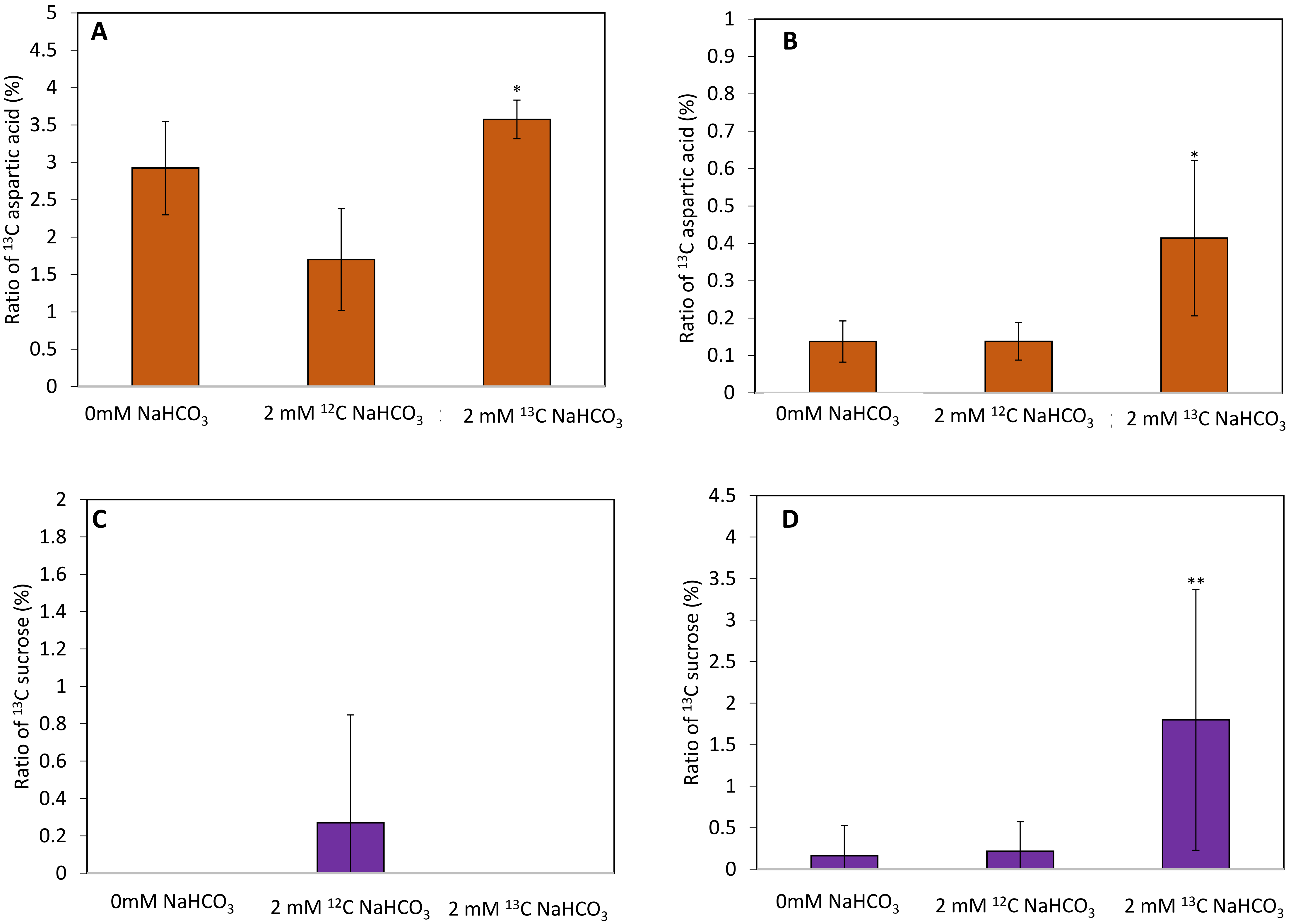

Supplementary Figure S10. The ratio in percentage of ^{13}C aspartic acid and sucrose in the phloem and xylem sap of plants treated with ^{12}C and ^{13}C $NaHCO_3$ as sources of inorganic carbon applied to the roots. A: ratio of ^{13}C aspartic acid in the xylem sap. B: ratio of ^{13}C aspartic acid in the phloem sap. C: Ratio of ^{13}C sucrose in the xylem sap. D: ratio of ^{13}C sucrose in the phloem sap. Each value represents the mean ± SD of n=6. Asterisks indicate statistically significant differences (p-value ≤0.05*, ≤0.01 **, ≤0.001 ***, Paired Student t-test).

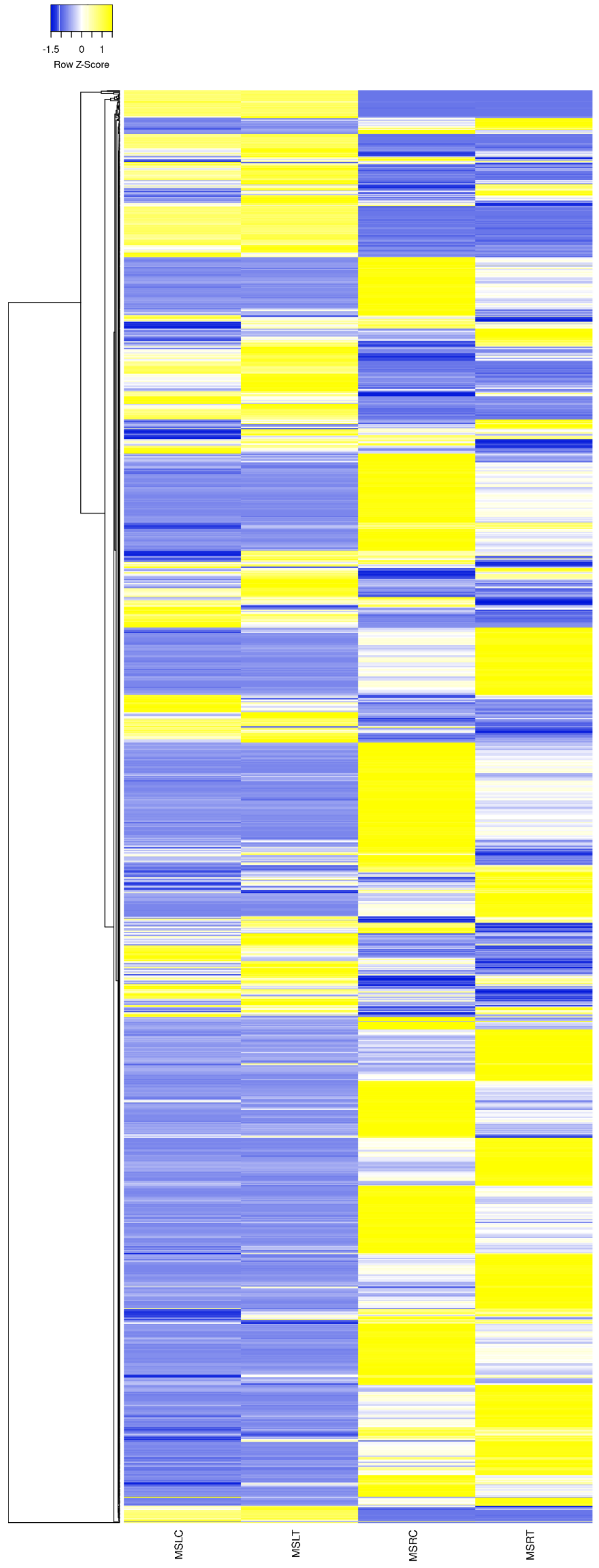

Supplementary Figure S11. Heatmap representing the DEGs of the comparison between the MSLC, MSLT, MSRC, and MSRT. For the heatmap the clusterization was performed through http://heatmapper.ca/. Using the z-score data transformation and Eucledian clustering by row. Values used for the graphic were the DEGs transformed to the FPKM values. The values were with a p-value of ≤ 0.05 and with a Log2 Fold change of ≥1**.** The yellow represents the upregulated DEGs and the blue the downregulated DEGs.

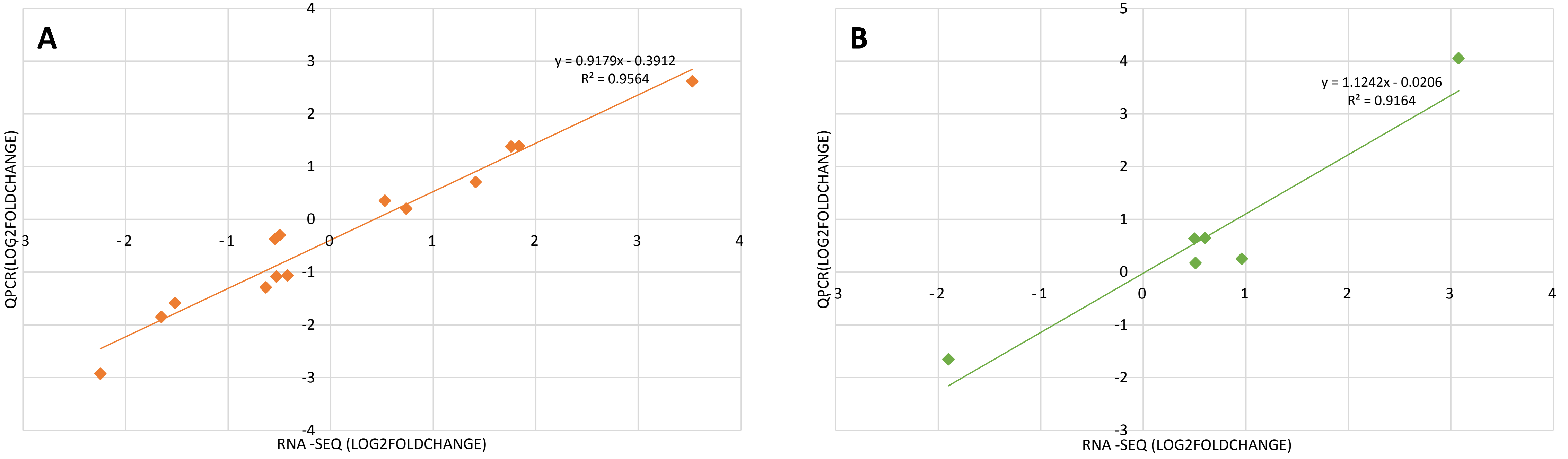

Supplementary Figure S12. Validation of the RNA seq data by qRT-PCR A. Validation of the RNA seq data of the MSRC vs MSRT by a correlation between the RNA seq and the qRT-PCR. B. Validation of the RNA seq data of the MSLC vs MSLT by a correlation between the RNA seq and the qRT-PCR.
